# Voltage-Driven Growth of Phosphorus Tribofilms

**DOI:** 10.1007/s11249-026-02173-6

**Published:** 2026-07-08

**Authors:** Yun Zhao, Jie Zhang, Hugh A. Spikes, Janet S. S. Wong

**Affiliations:** Department of Mechanical Engineering, Imperial College London, London, SW7 2AZ UK

**Keywords:** Electrified tribology, Phosphite, Phosphate, Tribofilm, Iron oxidation, Voltage

## Abstract

Ashless phosphorus-based lubricant additives, which are increasingly deployed in next-generation formulations, often suffer from slow tribofilm formation and poor film stability, limiting their effectiveness under demanding operating conditions. As mechanical systems become increasingly electrified, understanding how lubricants respond to electrical stimuli and developing strategies that exploit such stimuli have become critical for ensuring reliable operation. Here, we investigate the tribological performance of bis(2-ethylhexyl) phosphite (BEPite) in polyalphaolefin (PAO2) lubricated, electrified steel/steel contacts. Current is kept low to limit current-induced effects. Compared to an unbiased surface, BEPite produces thicker and denser tribofilms on anodic rubbing surfaces as voltage increases. This enhancement, however, is only observed when sliding is present. Chemical analysis reveals the presence of oxidised PO_3_^2−^-related species and abundant iron in the tribofilm. Static electrochemical cell experiments show surface oxidation is enhanced on anodic surfaces. This suggests voltage-driven triboelectrochemical oxidation and reaction promote tribofilm formation. Either iron oxides or released Fe ions may alter the tribofilm structure. These phenomena appear general for phosphorus-based ashless additives, as both phosphites and phosphates with different structures have seen increased tribofilm growth at anodic rubbing surface. This work demonstrates that an applied voltage, coupled with sliding, can intensify triboelectrochemical reactions, providing new insights for the design of next-generation lubricants.

**Supplementary Information:**

The online version contains supplementary material available at https://doi.org/10.1007/s11249-026-02173-6.

## Introduction

The rapid development of electrification has posed challenges to lubricated systems. For instance, battery systems in electric vehicles operate at 200–900 V, with voltages on motors reaching up to ~ 10% of the supply voltage and currents of tens of amperes. Current discharge can potentially lead to premature damage in EV drivetrain [1, 2]. The detrimental effect of electric potential has also been seen in wind turbine gearboxes and industrial motors [3]. Negative effects on performance of lubricant have also been reported [4–6]. Thus, a good understanding of lubricant behaviour under electrified conditions and potentially how it may be used to improve machine reliability is crucial.

Antiwear additives are important additives in fully formulated lubricants. Zinc dibutyldithiophosphate (ZDDP), the most common antiwear additive, has been a target of replacement due to component reliability and environmental issues [7–9]. Ashless phosphorus-based (P-based) additives, such as phosphates and phosphites, are potential candidates. These additives often suffer from limitations, including slow tribofilm growth and insufficient surface protection, making the enhancement of their performance a key research direction [10, 11]. Recent studies have focussed on molecular design strategies, including heteroatom substitution, carbon chain length, branching, and degree of esterification, to regulate adsorption and tribochemical reactions [12, 13]. In addition, synergistic effects in multi-additive systems have been explored to enhance film formation and tribological performance [14–16, 34]. Alternatively, external stimuli, such as applied electric fields, may offer a new route to promote tribofilm formation. In this work, we examine how an electrified contact may promote tribofilm formation of phosphite additives in the boundary lubrication regime.

## Background

Although lubricants with hydrocarbon oil as base fluid exhibit very low ionic conductivity, application of current and electric field can still affect lubricant performance. Numerous studies have shown that performance of fully formulated oils is influenced in electrified contact [6, 17–21]. For example, both wear volume in formulated engine oil and electric vehicle oil under 1 A and 2 A currents increased by 20–50 times, but only on the anodic surface, the cathodic surface remained unaffected [18]. A plausible explanation is that electric current is concentrated at the asperity contacts, generating significant localised heat that can potentially induce lubricant decomposition [5]. An interesting phenomenon has been demonstrated where the tribofilm formed on the anode becomes electrically polarised and subsequently adhered to the cathode [18]. Kadiric et al. [5] found that even a small current (< 10 mA) could affect tribofilm formation in an automatic transmission fluid, with tribofilm promoted on the anode but being suppressed on the cathode. Both studies highlight that electrifying a rubbing contact can alter the behaviour of additives, and hence, tribofilm formation and consequently affect the wear of tribo-pairs.

Additive performance may be impacted by electrochemical reactions even under mild voltage conditions. Under boundary lubrication, electrochemical reactions may be intensified at sliding interfaces due to shear stress. Most existing work to date about specific additive performance under electric field has focussed on film-forming additives such as zinc dibutyldithiophosphate (ZDDPs), molybdenum dithiocarbamate (MoDTC), and related chemistries [5, 17, 20, 22–26]. In 1981, Yamamoto and Hirano found that applying voltage and current simultaneously to rubbing pairs enhanced the tribofilm formation of tricresyl phosphate (TCP), whereas excessive voltage and current reduced it [27]. Ozimina later reported that 1 wt% ZDDP in acetonitrile could decompose at potentials within ± 1 V [24]. Applied voltages between 0 and 3 V have also been shown to influence additive adsorption and electrochemical reaction in ZDDP-containing propylene carbonate/diethyl succinate systems [28] and an organic molybdenum additive in PAO2 [29]. Wear reduction was observed as a result. For ashless additives, tricresyl phosphate has been reported to enhance the scuffing resistance of surface films under microampere‑current and millivolt‑voltage conditions [4]. By contrast, other studies that applied electrical current have shown detrimental effects on wear performance [18, 30].

An applied voltage may also impact tribofilm formation through altering the properties of rubbing surfaces. It has been shown that electrifying a contact could modify metal surface energy, thereby influencing frictional performance [31]. Applied voltage can affect metal surface oxidation [32], lubricant bubble generation [33], and base oil wettability [34]. Applying current can also induce similar phenomena described above, including surface interactions [27, 35] and bubble generation [36]. Prior work has shown that additive binding and tribochemical pathways differ between metallic iron and iron-oxide surfaces [37], and that iron cations generated due to metal surface oxidation can promote the polymerisation of iron polyphosphates near the substrate–tribofilm interface [38]. Additional studies have demonstrated that adding metal ions into lubricants enhances tribofilm formation [39], confirming the role of metal ions on tribofilm formation. From a triboelectrochemical perspective, promoting oxidation of the metal rubbing surface may facilitate the tribofilm formation of phosphorus-based additives, thereby improving their overall tribological performance. In this study, we control the voltage and current between lubricated contacts and investigate their influence on tribofilm formation of phosphite and phosphate additives to examine if voltage alone, without significant current, can modify their tribofilm formation. The chemical characteristics of the resulting tribofilms under different voltage biases are compared to evaluate the extent to which electrical stimuli modify interfacial chemistry. Complementary static electrochemical measurements, as well as tests in pure rolling, are performed to examine the importance of rubbing in the observed voltage-dependent tribofilm formation.

## Materials and Methods

### Materials

Cleaning solvents include heptane (≥ 99%) and toluene (≥ 99.5%). The lubricant additives used are bis(2-ethylhexyl) phosphite (96%), bis(2-ethylhexyl) phosphate (97%), dibutyl phosphite (96%), tris(2-ethylhexyl) phosphite, and tris(2-ethylhexyl) phosphate (97%), all purchased from Sigma-Aldrich without further purification. PAO2 (polyalphaolefin SpectraSyn 2) is chosen as the base oil. Its viscosity is 7.03, 3.90, 2.46, 1.66, and 1.21 mPa·s at 20, 40, 60, 80, and 100 °C, respectively, and its dielectric constant is approximately 2.

Lubricants are prepared by dissolving phosphorus-based additives into PAO2 to achieve a phosphorus concentration of 800 ppm, in accordance with the International Lubricant Specification Advisory Committee (ILSAC GF-6) standard for API engine oils [41]. The mixture is stirred with a magnetic stir bar at room temperature for 2 h. The transparent solution is then allowed to stand for 10 min and is used immediately thereafter.

Most of the results in this study use bis(2-ethylhexyl) phosphite (BEPite) in PAO2 as the model system. This is primarily because its 2-ethylhexyl group is a representative alkyl structure widely used in lubricant additives (in ZDDP systems), providing excellent compatibility and solubility in hydrocarbon base oils such as PAO2 [42, 43]. Under the present test conditions, BEPite forms a measurable tribofilm, allowing clear observation of voltage-induced effects.

### Tribometer and Test Conditions

**Fig. 1:**
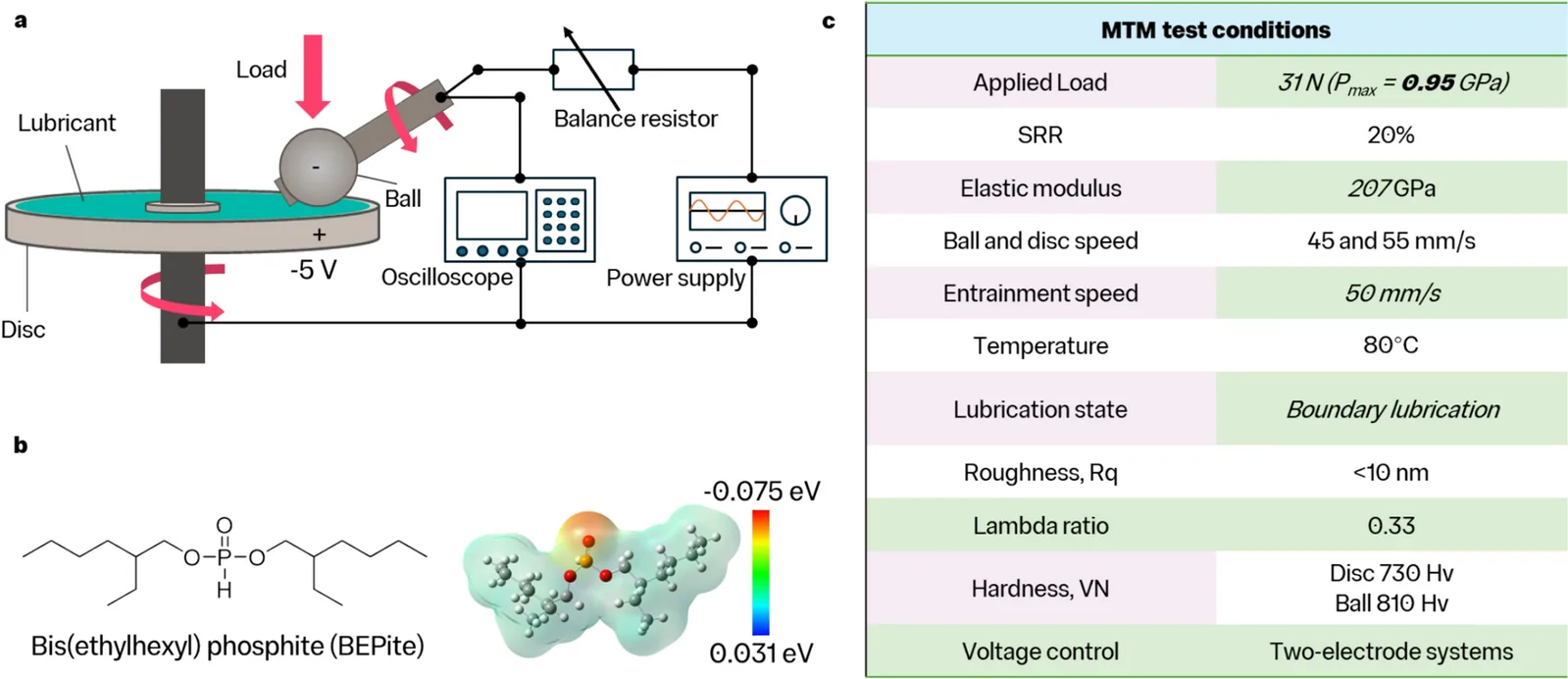
Experimental setup and testing conditions. **a** MTM-EC test rig equipped with voltage and current control system, which shows an application of −5 V via the power supply. **b** Chemical structure of bis(2-ethylhexyl) phosphite. **c** MTM testing conditions. Estimated lubrication film thickness and Lambda ratio are based on the Hamrock & Dowson equation (see Supporting Information S1) [40]

Friction tests are conducted using a mini-traction machine with an electrochemistry module (MTM-EC, PCS Instruments), as shown schematically in Fig. 1a. The MTM-EC employs a ball-on-disc configuration, with voltage applied across the ball and disc in a two-electrode setup. A balance resistor, with a maximum value of 1 MΩ, is connected to the contact in series and is used to control the electric circuit current. Voltage and current between the ball and disc are continuously monitored using an oscilloscope. The balls and discs are made of AISI 52100 steel and are from PCS Instruments. They are cleaned before testing using the following procedure: (1) rinse with toluene and wipe with toluene-soaked tissue; (2) ultrasonicate in toluene for 15 min, and then soak in fresh toluene for 12 h; (3) rinse again with toluene, wipe with toluene-soaked tissue, and ultrasonicate for another 15 min in fresh toluene; (4) before testing, wipe and rinse with toluene, dry with compressed air, and treat with oxygen plasma. The treatment is carried out using a Zepto One plasma system (Diener Electronic, Germany) under the following conditions: power of 30 W, treatment time of 1 min, oxygen atmosphere at a flow rate of 50 mL min^−1^.

Test conditions are summarised in Fig. 1b and c. The normal load is 31 N (maximum Hertzian pressure of 0.95 GPa), entrainment speed 50 mm s^−1^, and slide-roll ratio (SRR) 20%, with the ball and disc rotating at 45 and 55 mm s^−1^, respectively. Tests are performed at 80 °C. In the MTM-EC software, a + 5 V setting connects the positive terminal to the ball (anodic) and the negative terminal to the disc (cathodic). A − 5 V setting reverses this polarity. Unless otherwise stated, the balance resistor is set to 1 MΩ to minimise current effects. The measured potential of the contact is typically lower than the set value and may fluctuate. It is determined by the amount of solid-to-solid contact and electrical properties of any tribofilm formed on the surface. Thus, using contact voltage to label the tests may be misleading. All results are referenced to the applied voltage to ensure clarity. The test duration is 2 h, unless specified otherwise. After testing, specimens are rinsed with heptane and dried with compressed air. They are stored for further analysis. Each experiment condition is repeated at least twice to ensure reliability.

### Characterizations

After each test, wear scars may form on rubbing surfaces. Rubbed surfaces are first examined using an optical microscope to assess dimensions and colour of these scars (RH-2000 digital microscope, Hirox, Tokyo, Japan). The thickness of tribofilm on discs is measured using scanning white light interferometry (SWLI, Bruker Contour GT-K). The topographic profile is an average of a band of 5-pixel width. Before examining with SWLI, selected areas of the tribofilm on the disc are removed by etching with 0.1 M oxalic acid for 20 s to expose the buried steel surface. Residual oxalic acid is then wiped away using a DI water-wetted lint-free tissue. This is followed by gold coating on both etched and unetched regions (Fig. S1). This facilitates the evaluation of wear depth and the actual tribofilm thickness [39, 44, 45]. No noticeable corrosion or surface damage of the steel substrate is observed, see Fig. S11. Chemical analyses are conducted using energy dispersive X-ray spectroscopy (EDX, Tescan Mira) and time-of-flight secondary ion mass spectrometry (TOF–SIMS, PHI NanoTOF II). The testing region is 100 µm × 100 µm. EDX is used to map the element distribution in the tribofilm, while TOF–SIMS is employed to characterise the chemical states of elements at different depths.

## Results and Discussion

**Fig. 2:**
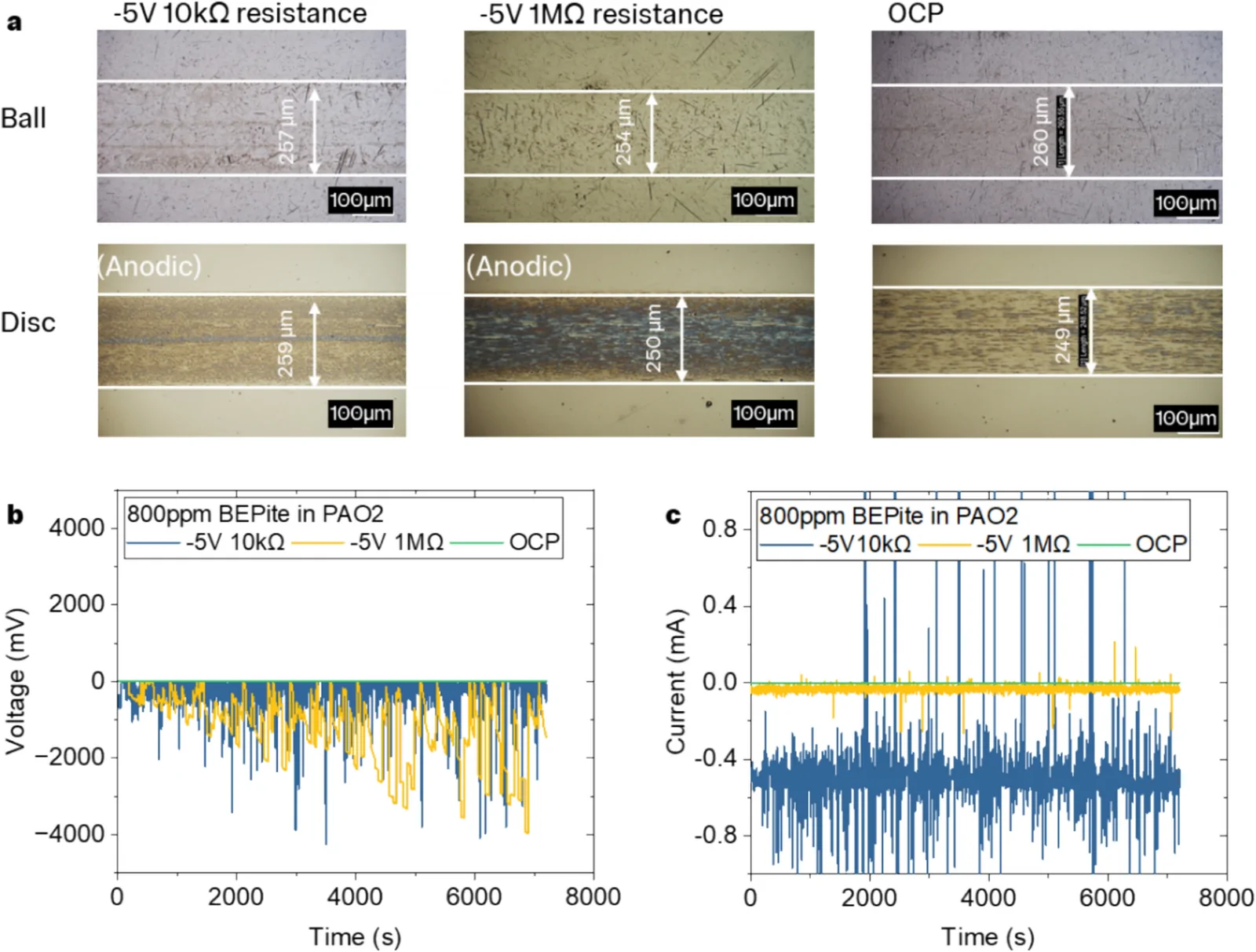
Worn surfaces formed from BEPite solutions under different current conditions. The external applied voltage is fixed at − 5 V. **a** Optical microscope images of the ball and disc after rubbing. The change in balance resistor from 1 MΩ to 10 kΩ corresponds to average voltage and current changing from 1.16 V and 0.01 mA to 0.26 V and 0.5 mA. The differences in tribofilm formation on the ball and the disc may arise from differences in ball–disc contact conditions, specific test parameters, or possible transfer of tribofilm from the ball to the disc surface. **b** Real-time voltage between rubbing surfaces (contact voltage) at different balance resistor values. **c** Real-time current between rubbing surfaces (contact current) at different balance resistor values. Data are averaged over intervals of 0.6 s

Under open-circuit potential (OCP) conditions, no external voltage is applied between the ball and disc. So, both voltage and current are negligible. In this state, the ball exhibits a wear track with no obvious tribofilm. On the disc, a patchy tribofilm, with low surface coverage, is formed (see Fig. 2a).

### Comparative Importance of Current and Voltage on BEPite Tribofilm Formation

In this study, the power supply provides a fixed voltage, while the impedance in the circuit varies. In addition to the fixed balance resistor, the resistance between the sliding contact is monitored and drops to 0 Ω when asperities of opposite surfaces are in direct contact, but becomes very high when the contact surfaces are separated by a tribofilm. As a result, the voltage and current across a sliding contact change and exhibit large fluctuations as the condition of the contact changes during rubbing. We record data continuously and then average the readings over each 0.6 s. Changing the resistance of the balance resistor from 1 MΩ to 10 kΩ corresponds to a change in the real average contact voltage and current from 1.16 V and < 0.01 mA to 0.26 V and 0.5 mA, respectively (Fig. 2b and c). The nominal current density, estimated based on the wear scar area (~ 250 µm diameter), is approximately 20 mA cm^−2^ and 1 A cm^−2^ for currents of 0.01 and 0.5 mA, respectively. The electrified conditions are kept mild to avoid current-induced damage. Such conditions enable the effects of voltage to be isolated, allowing mechanistic insights into its roles in tribofilm formation. To confirm, the electrical power dissipated through Joule heating is estimated to be approximately 58 mW (see Supporting Information S2). This is similar to the estimated frictional heating power of 34 mW, which results in a contact temperature rise of 2.1 K. Thus, the applied test conditions are unlikely to result in any substantial thermal effect within the contact.

A higher current combined with lower voltage (10 kΩ resistance) produces tribofilms similar to those formed under OCP conditions, whereas a lower current (1 MΩ resistance) combined with higher voltage results in a pronounced increase in tribofilm formation (Fig. 2a). These results confirm that voltage plays a more decisive role than current in driving tribofilm growth in our rubbing conditions. Thus, the following discussion focuses on the effects of applied voltage.

### Effect of Applied Voltage on BEPite Tribofilm Formation

**Fig. 3:**
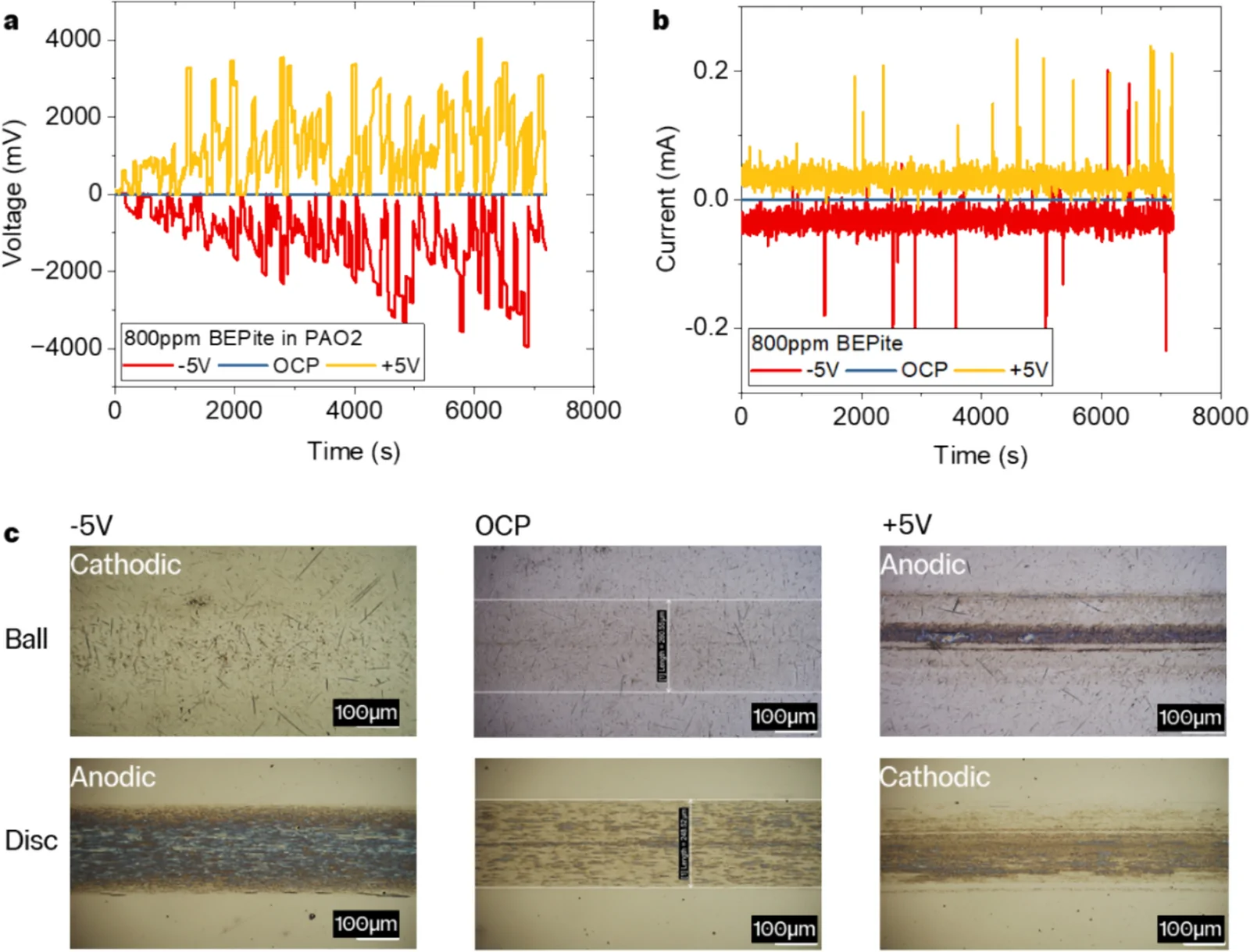
Effect of voltage on worn surfaces formed in BEPite solutions at a fixed balance resistance = 1 MΩ. **a** Real-time voltage between rubbing surfaces. **b** Real-time current between rubbing surfaces. Data are averaged over intervals of 0.6 s. **c** Optical microscope images of the ball and disc after rubbing

When a voltage is applied, the potential difference between the ball and disc increases gradually with rubbing time (Fig. 3a). At the start of sliding, substantial asperity contacts cause a short circuit, with measured voltage close to 0 V. As the tribofilm develops, the resistance across the contact increases, leading to a rise in the measured voltage. The morphology of the tribofilm leads to occasional micro-short-circuits, causing large voltage fluctuations. With the balance resistor in series, the contact current remains stable at ~ 0.01 mA (Fig. 3b).

The disc tribofilm formed under anodic condition is more uniform than that observed at OCP, whereas the ball wear track shows no obvious tribofilm (Figs. 3c**, **S3a, and S3b). For a cathodic disc, tribofilm is observed mostly at the centre of its wear track. Tribofilms are also formed at the centre of the wear track of the anodic ball. These observations indicate that tribofilm formation depends on the applied voltage and, critically, on its polarity. Notably, the friction coefficients under electrified and non-electrified conditions are similar (Fig. S3c–S3f). Since the asymmetric geometry of our rubbing contacts may impact on the interpretation of polarity effect, the tribofilm on the disc is selected as the focus for subsequent analysis to enable a more reliable assessment of the effect of applied voltage on tribofilm formation.

**Fig. 4:**
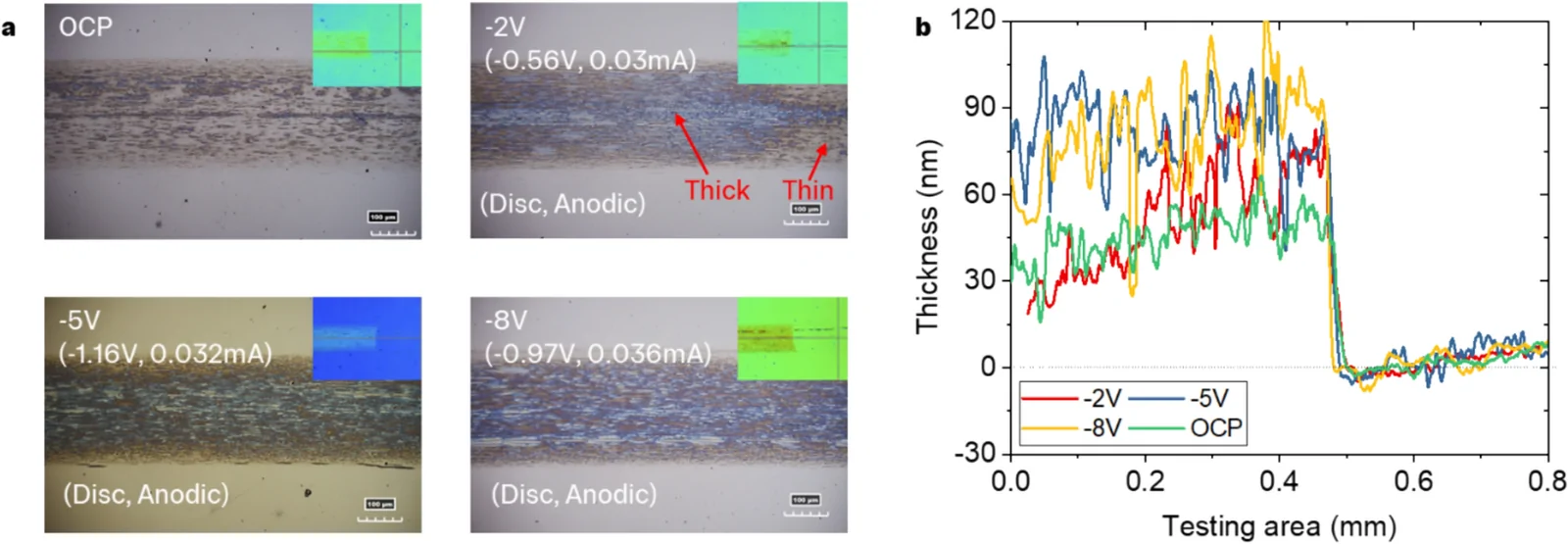
BEPite tribofilms formed under different voltage conditions. **a** Optical microscope images of anodic discs after 2-h rubbing under different applied voltages. Inset SWLI images show etched tribofilm for thickness measurement. White scale bars correspond to 100 µm. **b** Thickness of tribofilm along the rubbing direction formed under different voltages. Repeat tests showing profiles parallel and perpendicular to the rubbing direction are in Fig. S4

The effect of voltage on tribofilm formation on an anodic disc is investigated over a range of applied negative voltage while keeping the balance resistor at its maximum value to minimise current effects, see Figs. 4a and S4. For set voltages of − 2 V, − 5 V, and − 8 V, the actual average contact voltages are − 0.56 V, − 1.16 V, and − 0.97 V, respectively (Fig. S5). At a set voltage of − 2 V, blue deposits (labelled as ‘thick’ in Fig. 4a) appear on the disc surface, with localised increase in thickness but poor uniformity. At − 5 V, tribofilm growth is enhanced, with more blue regions and an average thickness of 80–90 nm (Fig. 4b, and for repeat tests see Figs. S4 and S6). Further increasing the set voltage to − 8 V does not increase thickness. As contact voltage is governed by contact conditions, similar contact voltage between − 5 and − 8 V implies similar tribofilm conditions, which is indeed the case (Figs. 4a, S4, and S6e). In contrast, applying a positive voltage does not lead to increase in tribofilm thickness but can impact the morphology of the tribofilm (Fig. S7).

The increased thickness of tribofilms on anodic discs shows that an anodic bias results in higher tribofilm growth rate or stronger tribofilm (resisting film removal) or both. Note that the profiles of the cleaned area show no measurable wear, although occasional surface scratching can occur during the process (Fig. S8). This suggests that tribofilms formed in anodic discs under the applied negative voltage offer good antiwear protection.

**Fig. 5:**
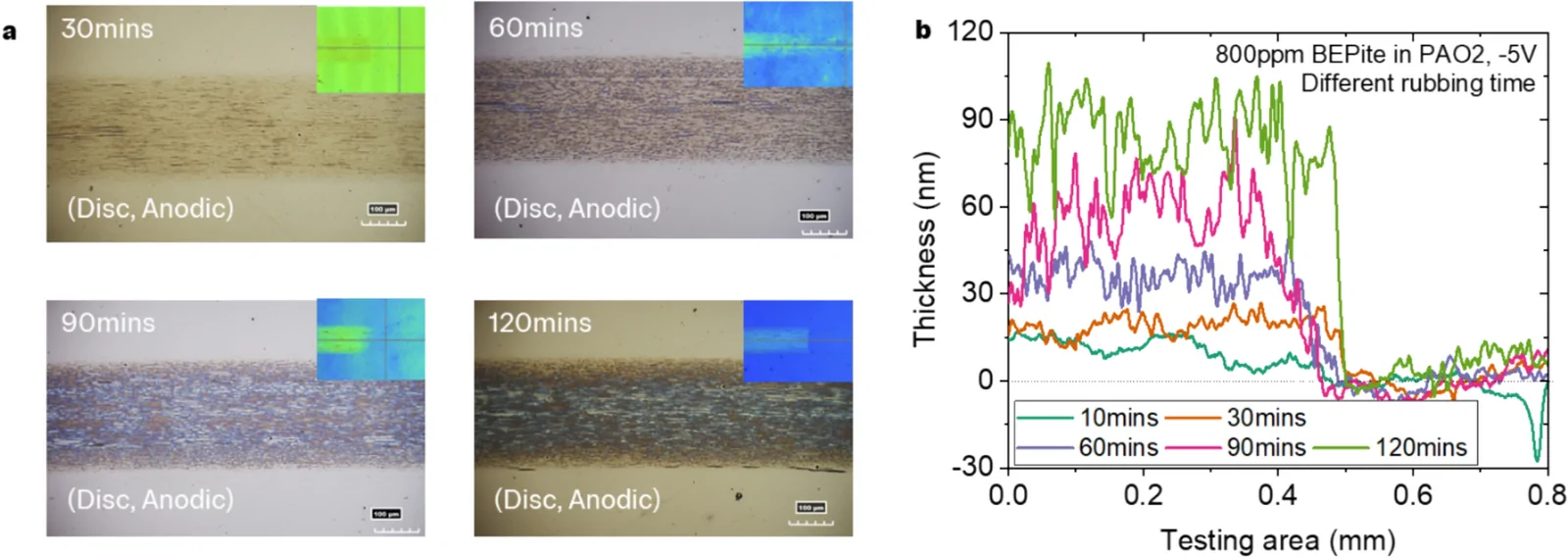
BEPite tribofilm on anodic discs over different rubbing durations under a set voltage of − 5 V (contact voltage: − 1.16 V). **a** Optical microscope images of the disc after different rubbing durations under − 5 V. White scale bars correspond to 100 µm. Inset images show etched tribofilm for thickness measurements. **b** Thickness of tribofilms along the direction of rubbing, formed under − 5 V at different rubbing durations. For film thickness along the direction orthogonal to rubbing, see Figs. S9–S13

Figure 5 shows how the tribofilm thickness on anodic discs at − 5 V changes with rubbing time (see also Fig. S9a). In the first 30 min, the tribofilm islands reach only 15–20 nm, and they scatter across the wear track (see also Figs. S10 and S11). By 60 min, the thickness increases to ~ 40 nm (see also Fig. S12), and the surface becomes densely covered with granular and elongated deposits. Further rubbing produces thicker tribofilms, reaching ~ 60 nm at 90 min (see also Fig. S13) and ~ 80 nm at 120 min.

**Fig. 6:**
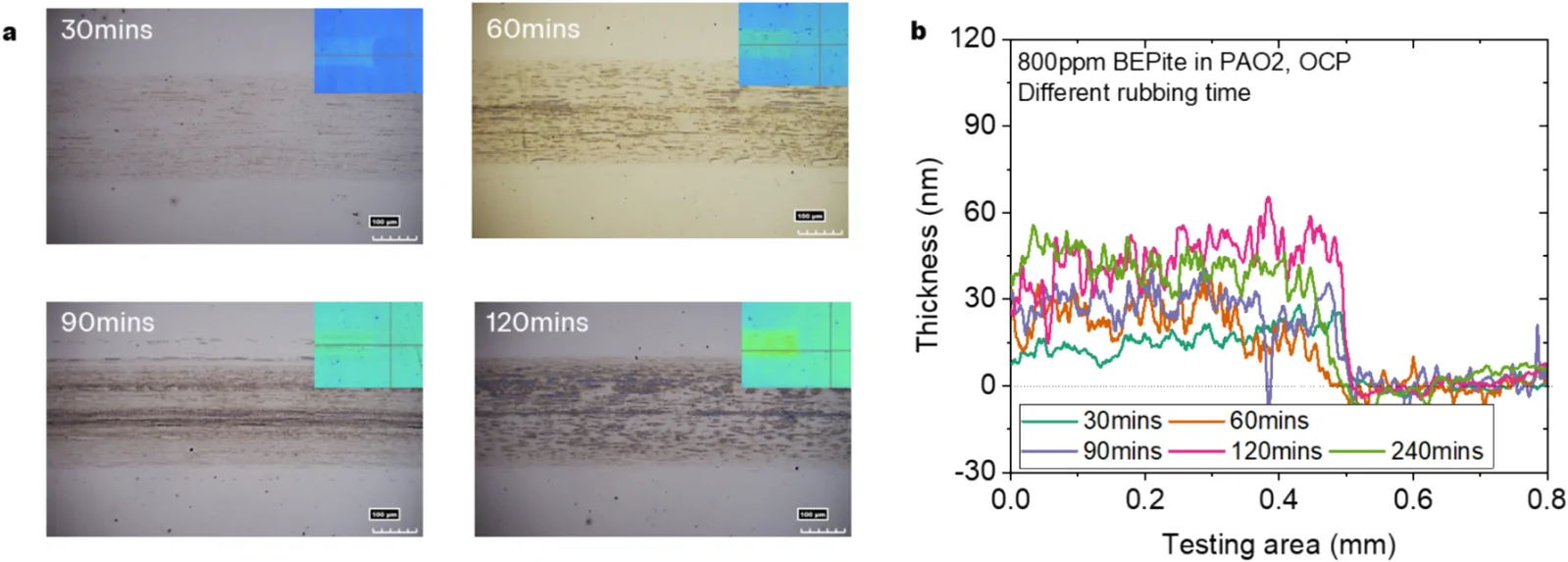
Frictional performance of BEPite in PAO2 over different rubbing durations under OCP. **a** Optical microscope images of the disc over different rubbing durations under OCP. White scale bars correspond to 100 µm. Inset images show etched tribofilm for thickness measurement. **b** Thickness of tribofilm along the direction of rubbing formed under OCP at different rubbing durations. For film thickness along the direction orthogonal to rubbing, see Figs.S14–S17

Tribofilm growth under unelectrified (OCP) conditions is slower (Figs. 6**, **S9b, and S14–17). After 30 min, the film thickness is ~ 15 nm (Fig. S14), with fewer deposits compared to that on an anodic disc under − 5 V. Prolonged rubbing increases tribofilm thickness marginally. After 120 min, it reaches only ~ 40 nm, with partial surface coverage. Extending the rubbing time to 240 min does not lead to further growth of the tribofilm thickness (Fig. S17). These results show that the effect of voltage can influence tribofilm formation even at the earliest stages. It increases tribofilm growth rate or produces more robust films that remain stable at higher thicknesses, or both.

### Chemical Analysis of Disc Tribofilm Formed at OCP and − 5 V

**Fig. 7:**
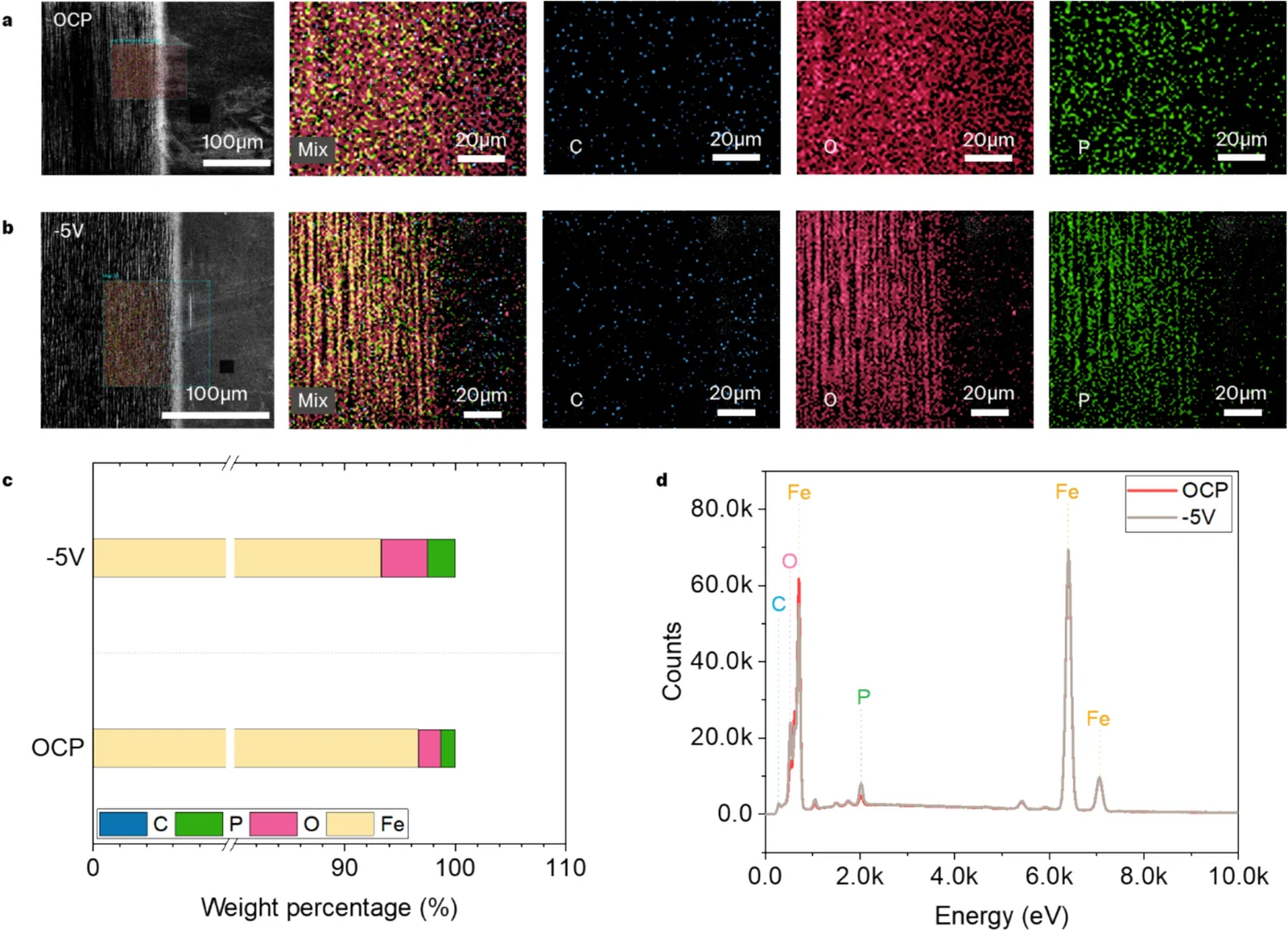
EDS analysis of tribofilm formed on the disc at OCP and − 5 V. **a** Element mapping of the tribofilm formed at OCP. The map area is shown in the SEM image (left). **b** Element mapping of the tribofilm formed at − 5 V. The map area is shown in the SEM image (left). **c** Weight percentage of various elements in the tribofilm. d. EDS spectra of tribofilms formed at OCP and − 5 V (contact voltage: − 1.16 V). Note the colour map in **a** and **b** are for elemental distribution only

Chemistry of tribofilms formed under electrified and non-electrified conditions is investigated using EDS, see Fig. 7. For direct comparison, the selected regions include both the wear tracks and the immediate unrubbed areas. In both cases, oxygen (O, red) and phosphorus (P, green) are detected. In non-electrified condition (Fig. 7a), the distributions of O and P in the wear track are slightly elevated. In the electrified condition (Fig. 7b), the distributions of O and P form stripe-like features which match the tribofilm morphology, showing that this tribofilm consists of O- and P-containing compounds.

The wear track on the anodic disc contains 2.52 wt.% P and 4.17 wt.% O, nearly twice the proportions observed on an unelectrified disc (Fig. 7c and d). On the other hand, the unelectrified disc contains more Fe. This result is consistent with tribofilms on anodic discs being thicker, and having higher surface coverage (see Fig. 4).

**Fig. 8:**
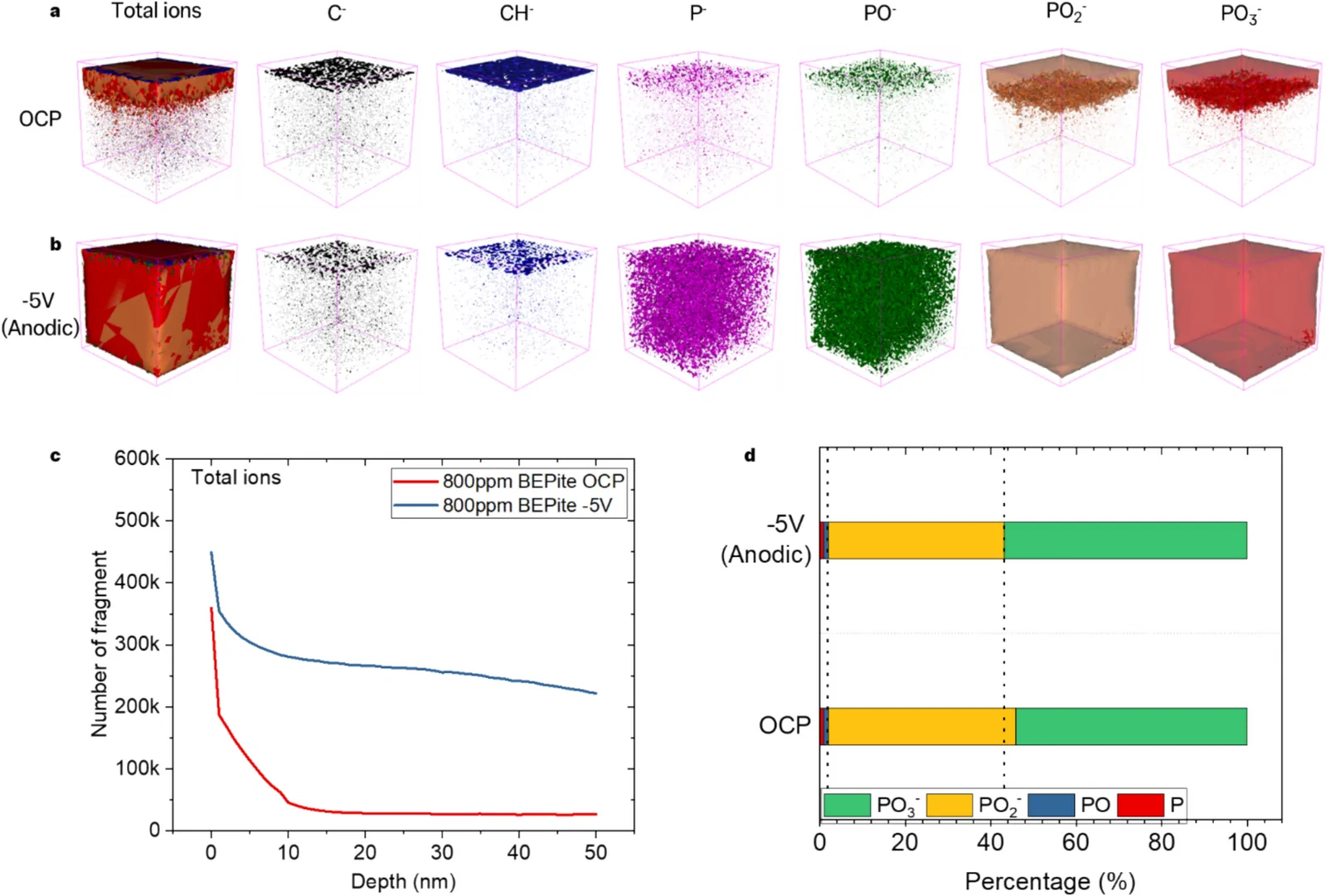
TOF–SIMS depth profiling of tribofilm formed on the disc at OCP and − 5 V. 3D distributions of negative ion fragments in the top 50 nm of tribofilms formed **a** at OCP, and **b** at − 5 V (contact voltage: − 1.16 V). **c** TOF–SIMS depth profiles of total negative fragments. **d** Percentage of P-based negative ion fragments

3D TOF–SIMS (Fig. 8) is performed in a ~ 100 μm × 100 μm area at the centre of each wear track (wear track width ~ 250 μm) at 10 nm increments for a total of five cycles. As phosphite-derived tribofilms typically contain C, O, and P, the negative ion spectra are analysed for C^−^, CH^−^, P^−^, PO^−^, PO_2_^−^, and PO_3_^−^ fragments. Phosphorus in the tribofilms on both discs is primarily present as PO_2_^−^ and PO_3_^−^, with a small amount of other P-containing fragments (P^−^ and PO^−^) detected (Fig. 8d). Overall, PO_3_^−^ and PO_2_^−^ account for 57 and 41% of total P-containing fragments in the anodic tribofilm, whereas in the OCP tribofilm, the proportions are 52 and 44%, respectively. No PO_4_^−^ (the oxidation product of phosphite) forms under OCP conditions, while it is detectable in anodic tribofilm, suggesting that anodic polarisation can drive this oxidation reaction (Fig. S18).

**Fig. 9:**
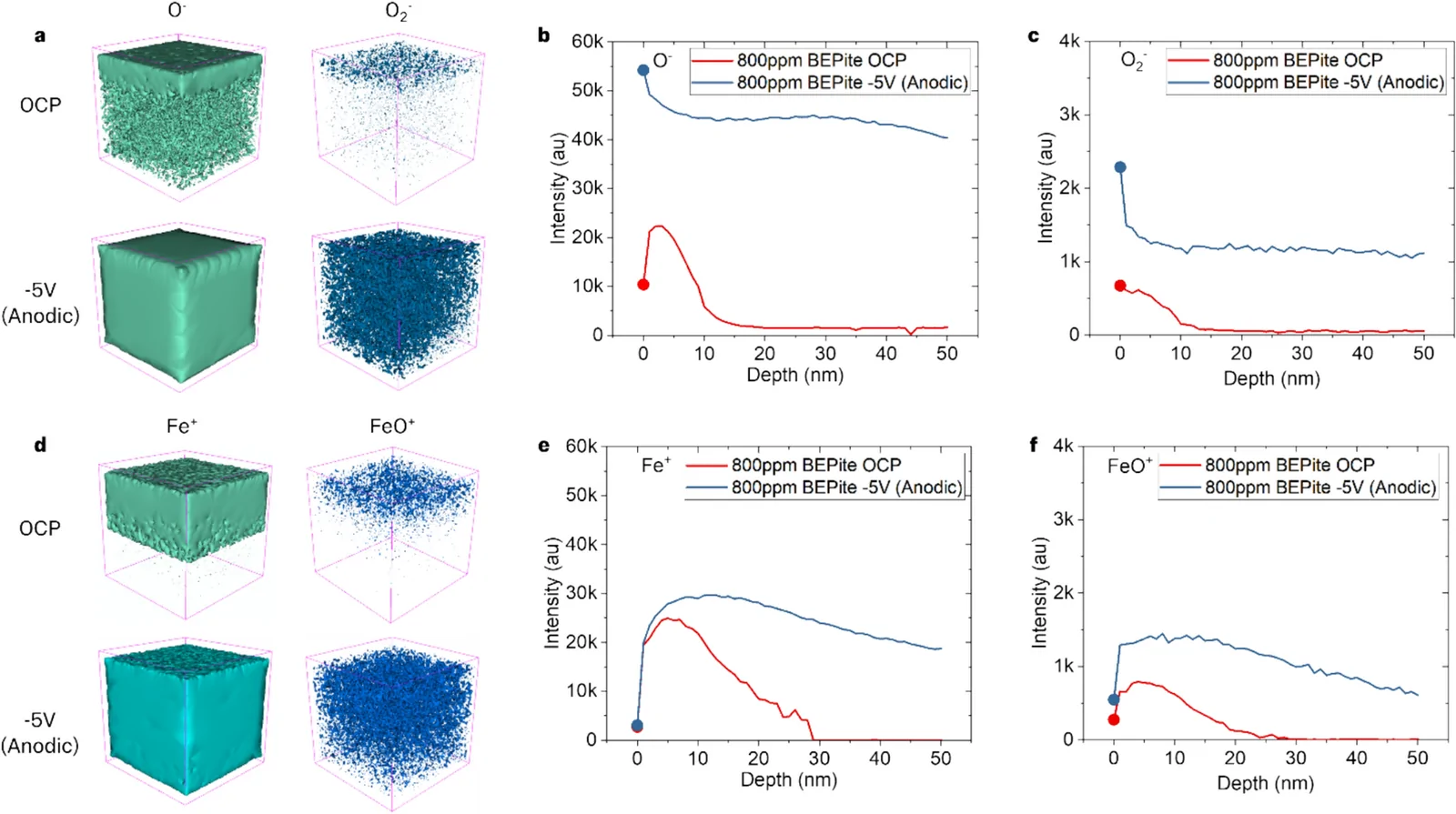
TOF–SIMS depth profiling of iron oxide in the tribofilm formed on the disc at OCP and − 5 V (contact voltage: − 1.16 V). **a** 3D tomography of O^−^ and O_2_^−^. **b** TOF–SIMS depth profiles of O^−^. **c** TOF–SIMS depth profiles of O_2_^−^. **d** 3D tomography of Fe^+^ and FeO^+^. **e** TOF–SIMS depth profiles of Fe^+^. **f** TOF–SIMS depth profiles of FeO^+^

The distribution of iron oxides in the tribofilm is also examined, as surface oxidation may be an important contributing factor to tribofilm formation. The key markers for iron oxides in TOF–SIMS includes Fe^+^, FeO^+^, FeOH^+^, Fe_2_O^+^, O^−^, O_2_^−^, FeO_2_^−^, and FeO_2_H^−^. Among these, only O^−^, O_2_^−^, Fe^+^, and FeO^+^ exhibit obvious signals (Fig. 9).

**Fig. 10:**
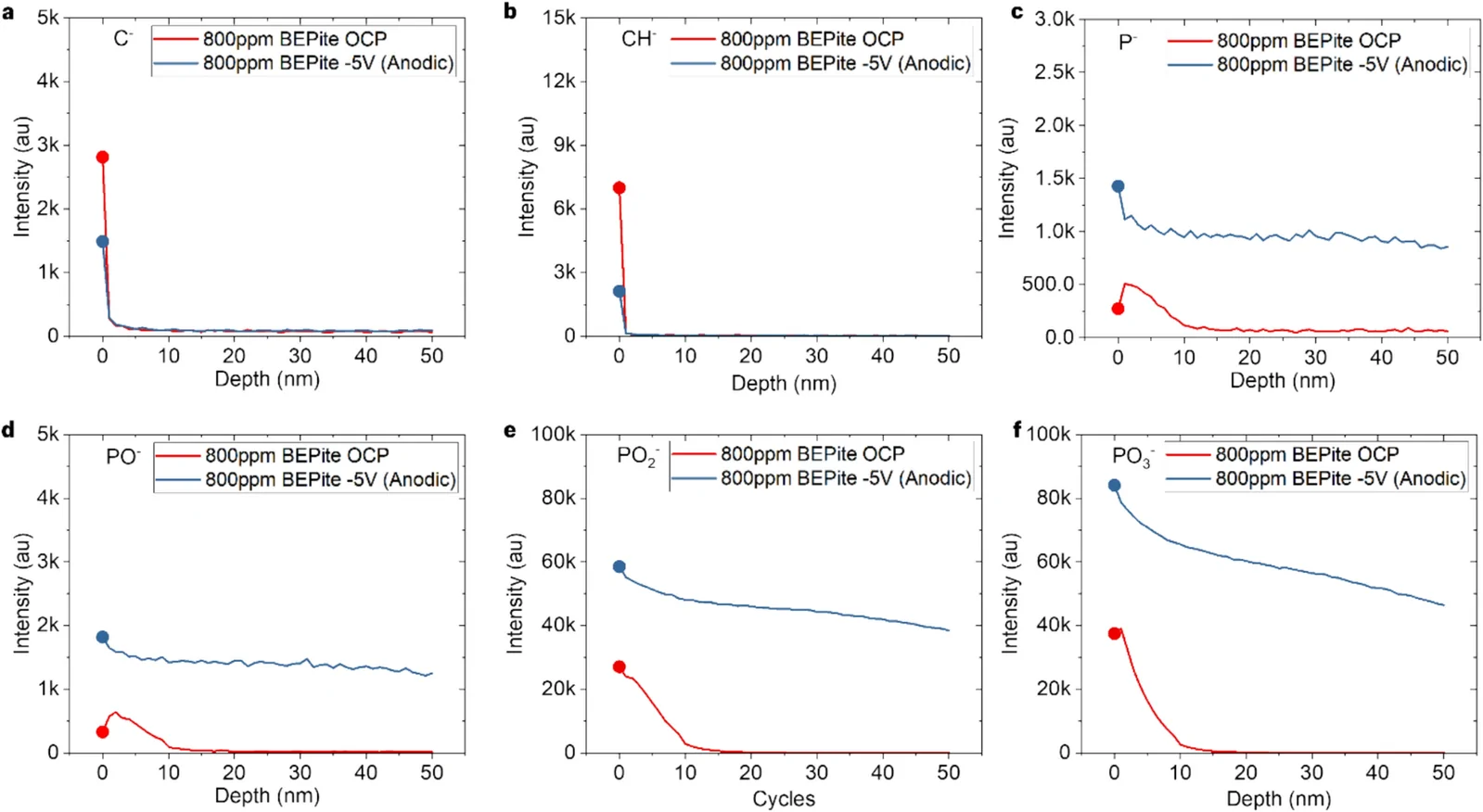
Negative ion TOF–SIMS depth profiles of individual fragments in the tribofilm formed on the disc at OCP and − 5 V (contact voltage: − 1.16 V). **a** C^−^. **b** CH^−^**c** P^−^. **d** PO^−^. **e** PO_2_^−^. **f** PO_3_^−^

Depth profiling confirms that the tribofilms formed under OCP are much thinner than those formed in − 5 V. The depth distributions and relative intensities of various fragments are shown in Figs. 8, 9, and 10. C^−^ and CH^−^, representatives of organic and carbonaceous species, are concentrated within the top 1–2 nm, consistent with previous reports [46], and are more abundant in the OCP tribofilm than in the anodic tribofilm (Figs. 8a and b and 10a and b).

The tribofilms on both the OCP and the anodic discs show the highest P-base fragment densities on their top surfaces. For the anodic tribofilm, the amount of P-based fragments drops within the first few nanometers before stabilising or reducing at slower rates. The high fragment concentration detected at the tribofilm surface probably originates from recent deposition and surface reactions during sliding. With increasing depth, these newly formed species may undergo further changes, such as organic species removal and inorganic species ageing, leading to the formation of a more stable tribofilm which is more resistant to rubbing. Depth profiles of iron-oxide fragments show high intensities across the entire sputtering range. They co-exist with P-base fragments throughout the tribofilm, indicating a stable, iron-phosphorus-rich structure. These results demonstrate that iron actively participates in tribofilm formation, leading to the development of a dense, iron-phosphorus-enriched tribofilm under anodic conditions. These depth profiles show that the through-thickness chemistry of the anodic tribofilm is relatively constant with the amount of phosphorus and iron-oxide-related species decays slowly below the top surface (Figs. 9 and 10).

The intensities of P-base fragments are much lower in OCP tribofilms and are only detected in the first 10 nm (Fig. 10). Iron oxide-related fragments concentrate in the top ~ 10 nm and gradually decrease in abundance at depths of 20 − 30 nm, consistent with a previous report about iron-oxide distribution within the tribofilm [47]. This exceeds the depth range of phosphorus-containing species, suggesting a tribofilm structure comprising an organic/carbonaceous top layer, a middle layer containing phosphorus and iron, and an underlying oxide layer.

### How Anodic Potential Promotes Tribofilm Formation

Under open-circuit potential (OCP), the tribofilm contains a high proportion of partially decomposed phosphite species, resulting in an organic-rich film. In contrast, anodic polarisation favours more complete phosphite decomposition, producing a tribofilm enriched in inorganic phosphate species $\left({PO}_{3}^{-}\right.$, ${PO}_{2}^{-}$, $\left.{PO}_{4}^{3-}\right)$ that better stabilise the film under shear.

**Fig. 11:**
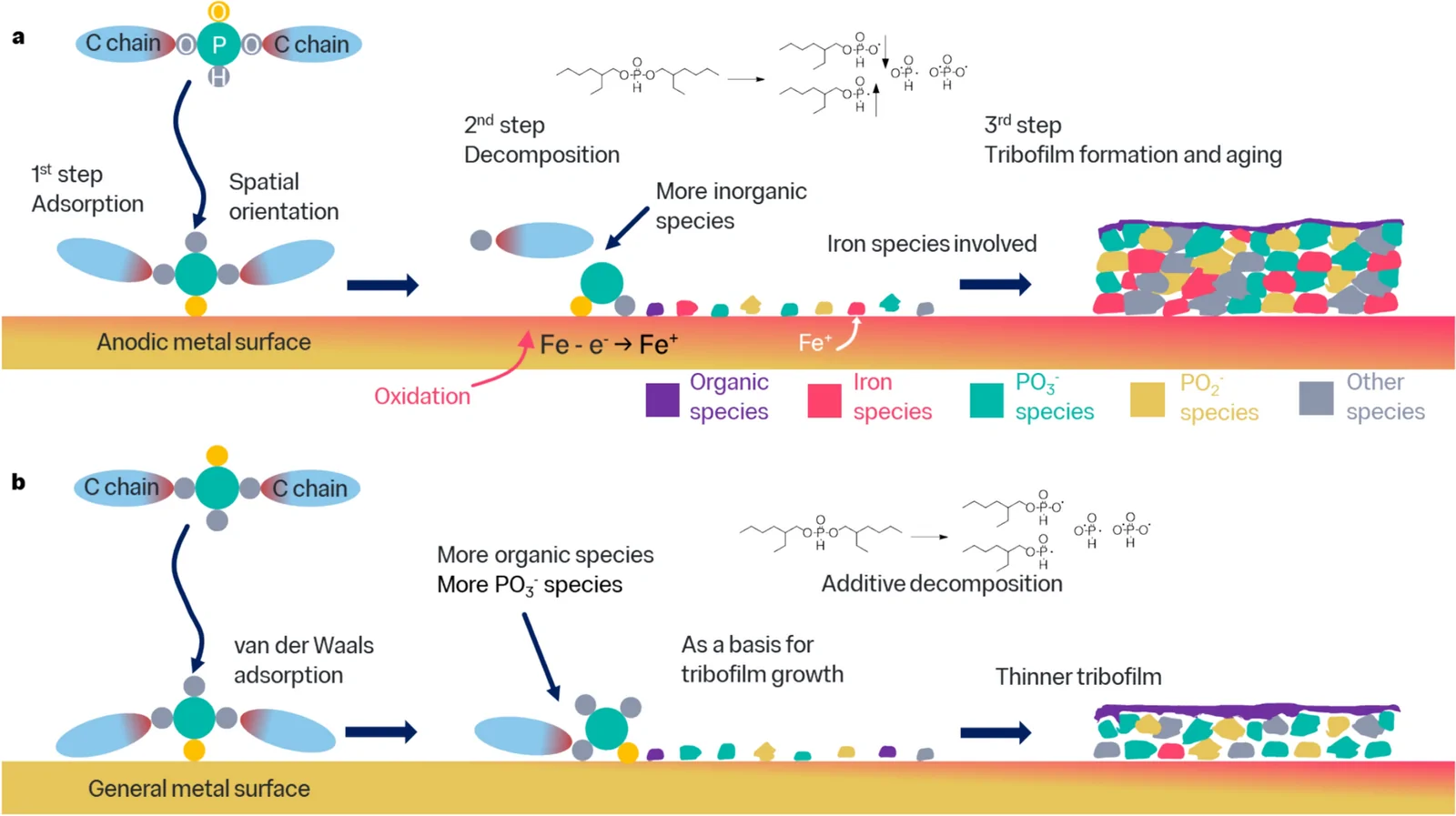
Schematic illustration of tribofilm formation on a steel surface under **a** OCP and **b** anodic potential conditions

**Fig. 12:**
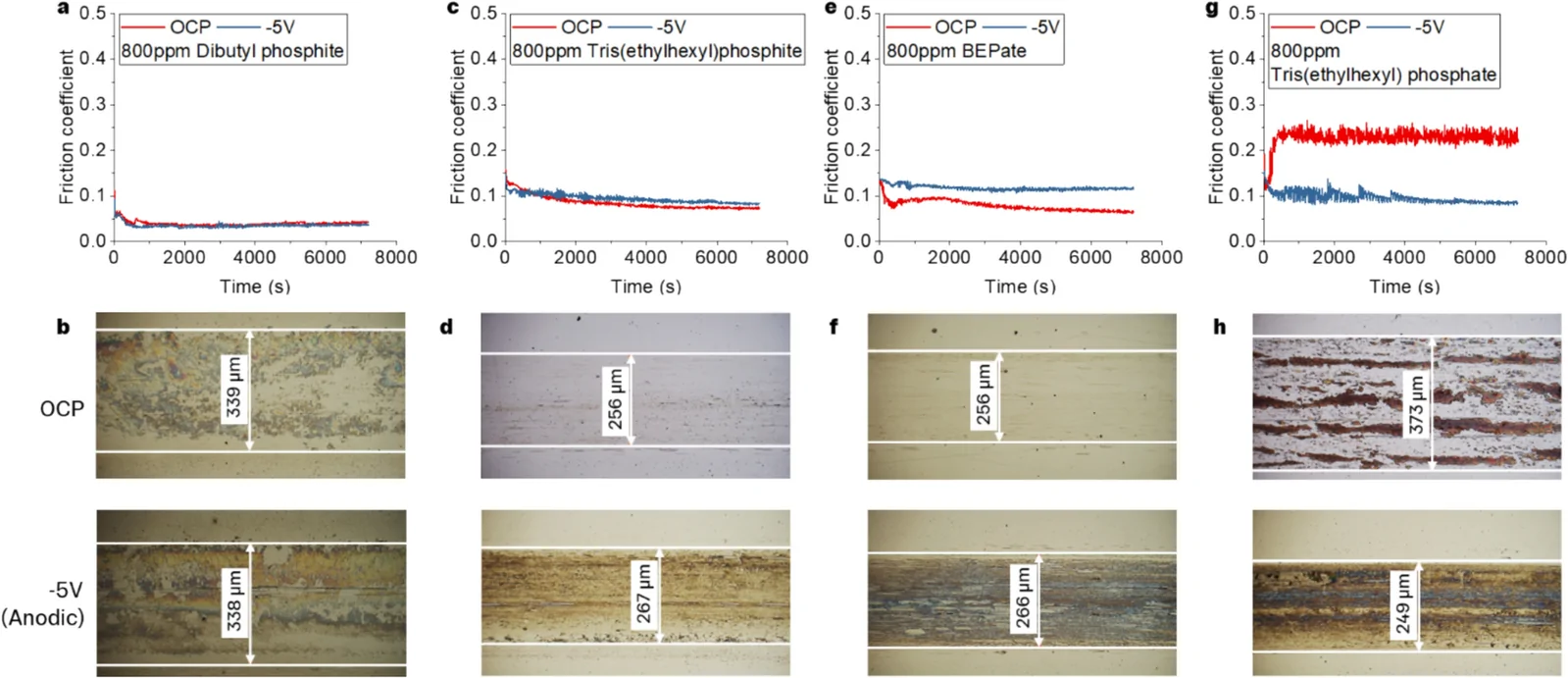
Frictional performance of phosphite and phosphate of various molecular structures in PAO2. COF results and wear track of **a** and **b** dibutyl phosphite; **c** and **d** tris(ethylhexyl) phosphite; **e** and **f** bis(2-ethylhexyl) phosphate (BEPate); **g** and **h** tris(ethylhexyl) phosphate

A potential mechanism for the formation of the tribofilm under applied anodic potential compared to OCP conditions is illustrated in Fig. 11. Phosphite additives adsorb onto the steel surface through chemisorption via the electronegative phosphorus group [13], assisted by physical interactions (van der Waals forces) between the organic carbon chains and the surface. When an anodic potential is applied, the resulting electrostatic field may strengthen chemisorption while concurrently weakening the physical adsorption of the organic chains because of electronegativity difference [48, 49], which may impact the initial adsorption and subsequent reactions. Cleavage of the C–O bond, as generally seen with ZDDPs [50], may favour the formation of PO_3_^−^, whereas scission of the O–P bond may lead predominantly to PO_2_^−^. The anodic film also contains PO_4_^−^. The possible weakened interaction between carbon chains and the surface may lead to reduced carbon species on rubbing surfaces (Fig. 12).

**Fig. 13:**
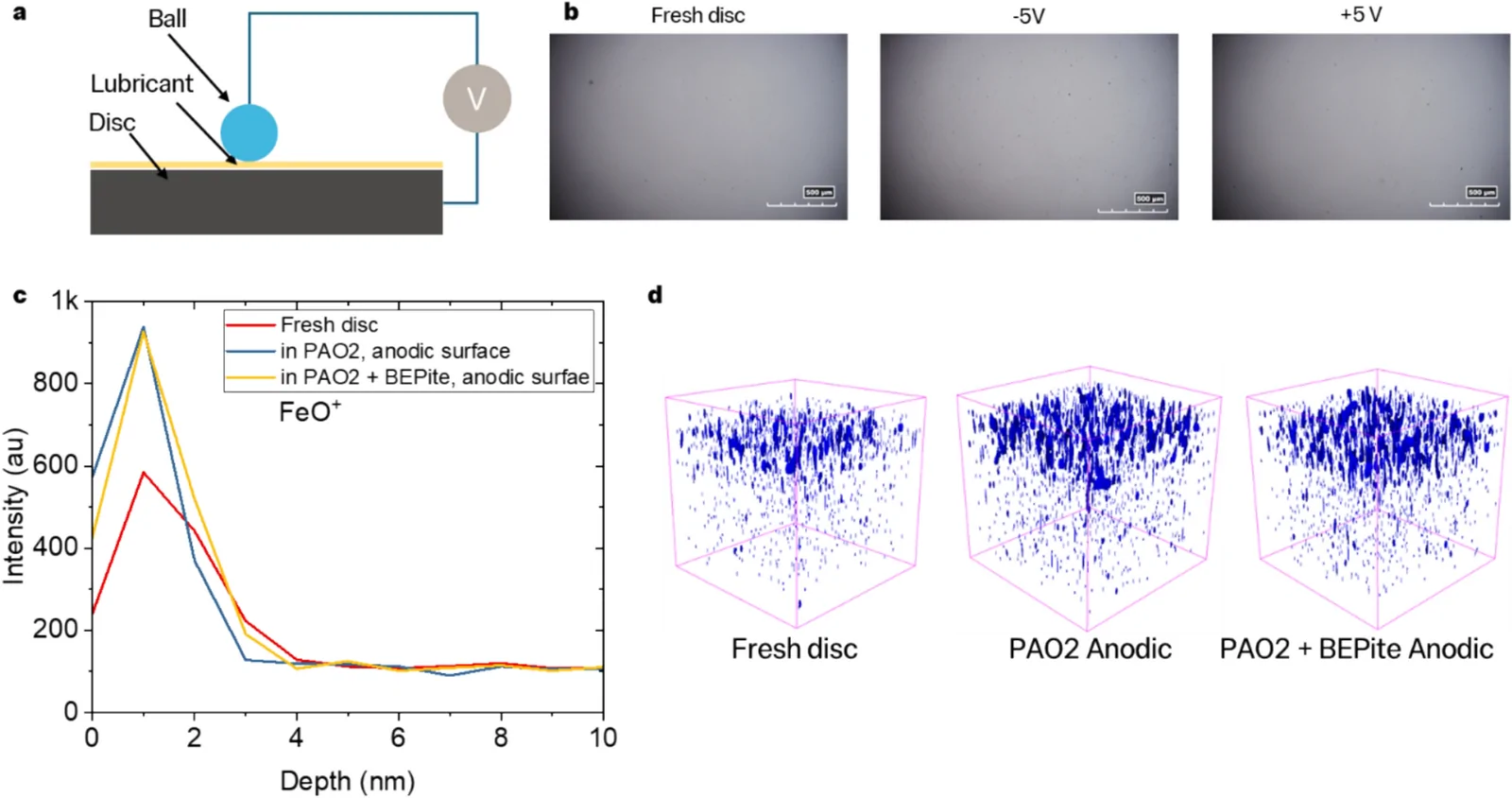
Electrochemically driven interfacial oxidation under static conditions. **a** Schematic of the setup used to probe static electrochemical reactions in which a steel ball and a steel disc are held in close proximity in a lubricant under an applied voltage. **b** Optical images of the steel disc before and after applying 5 V between ball and disc with 800 ppm BEPite in PAO2 for 4 h. Scale bars correspond to 500 μm. **c** TOF–SIMS depth profiles of FeO^+^ fragments of a fresh disc and an anodic disc. **d** 3D TOF–SIMS tomographic reconstruction of FeO^+^ distribution of discs in **c**

Anodic polarisation may also promote oxidation of the steel surface (see Fig. 13c), leading to the formation of iron oxides and the release of iron ions. In contrast, reduction processes at the cathodic surface are less likely to generate iron species. This difference may explain why thicker tribofilms tend to form on anodic surfaces. The presence of iron species may enhance both the chemisorption and complexation of phosphite derivatives, thereby accelerating tribofilm growth. The incorporated iron ions may play a vital role, acting as cross-linking centres, connecting phosphate groups to form a more coherent and robust three-dimensional network within the tribofilm [48]. This iron-stabilised architecture may significantly improve the mechanical integrity and shear resistance of the film under tribological stress.

The overall chemical nature of the OCP and anodic tribofilms observed here is consistent with tribofilms and thermally formed films reported in the literature [46, 51]. The films appear to be dominated by a mixture of carbon-containing species, phosphate-based compounds, and iron-phosphate/iron-oxide complexes, with an oxide-rich interfacial layer adjacent to the steel substrate. The key distinction due to the applied anodic potential therefore lies not in the identity of the chemical species, but in their relative abundance, distribution, and degree of inorganic cross-linking. Under anodic polarisation, the tribofilms tend to exhibit a higher fraction of inorganic phosphate species and iron-coordinated structures, whereas films formed under OCP or purely thermal conditions tend to retain a larger proportion of organic fragments from incomplete phosphite decomposition. This suggests that anodic polarisation does not necessarily introduce new chemistry, but rather shifts the balance of existing tribochemical pathways, promoting oxidation, enhanced iron incorporation, and the formation of a more inorganic, mechanically robust network compared with conventional phosphite tribofilms reported in the literature.

### Universality of the Applied Potential Effect on P-Based Tribofilm

The above effect of applied potential on promoting tribofilm growth is not unique to BEPite. Voltage-induced effects on tribofilm formation are also seen for other phosphorus-containing additives that show limited tribofilm growth under OCP conditions (Fig. 12). For example, diisopropyl phosphite forms tribofilm rapidly at OCP, and so applying voltage produces minimal effect (Fig. S19). In contrast, dibutyl phosphite, tris(2-ethylhexyl) phosphite, bis(2-ethylhexyl) phosphate, and tris(2-ethylhexyl) phosphate, which all show limited tribofilm formation at OCP, show obvious changes in their tribofilms under − 5 V. For dibutyl phosphite, friction coefficient remains unchanged, while tribofilm thickness slightly increases under − 5 V (Fig. 12a and b). Tris(2-ethylhexyl) phosphite (Fig. 12d) and bis(2-ethylhexyl) phosphate (Fig. 12f) produce no obvious tribofilm at OCP, but tribofilms are formed at − 5 V, accompanied by an increase in friction coefficient (Fig. 12c and e). Tris(2-ethylhexyl) phosphate (Fig. 12h and g) shows large differences in tribofilm morphology and friction coefficient between OCP and − 5 V. At OCP, the wear track is wider and tribofilm highly heterogeneous, while under − 5 V the wear track becomes narrower and the tribofilm more uniform, corresponding to a reduction in friction coefficient from 0.25 to 0.10. These results indicate that applying a negative potential can promote tribofilm formation in a variety of P-based additives.

### Effect of Rubbing

One question that arises from the above tests is whether the observed effect on BEPite tribofilm on anodic discs in a rubbing contact can occur without rubbing. To explore this, the growth of the voltage-induced surface film is evaluated with (1) a static electrochemical cell, as well as (2) in a MTM-EC tribometer under pure rolling condition.

A static electrochemical cell is created with a steel ball and a steel disc brought into close proximity in a lubricant. The separation between the two surfaces is controlled at approximately 1 μm using a digital micrometer (Fig. 13a). In the absence of mechanical motion, electrochemical effects are isolated from tribochemical effects.

Applying a 5 V bias to steel discs in BEPite in PAO2 for 4 h leads to no obvious surface film under optical microscopy (Fig. 13b). Raman spectroscopy likewise reveals no discernible signatures of iron oxides, suggesting that any oxidation, if present, occurs at very low levels (Fig. S20). More sensitive TOF–SIMS depth profiling shows only a slight increase in FeO^+^ fragments on the anodic surface after holding at − 5 V for 4 h, both in neat PAO2 and in PAO2 containing 800 ppm BEPite (Fig. 13c and d). Carbon‑rich residues are also observed, likely due to the adsorption of tenacious carbon (Fig. S21). The disc immersed in a phosphite‑containing PAO2 solution has a thin PO_2_^−^- and PO_3_^−^-containing layer, which adheres strongly and cannot be removed by rinsing with heptane (Figs. S21 and S22). This enhancement is confined to the top ~ 1–2 nm of the interface, indicating extremely limited electrochemical oxidation and surface film formation under static conditions.

**Fig. 14:**
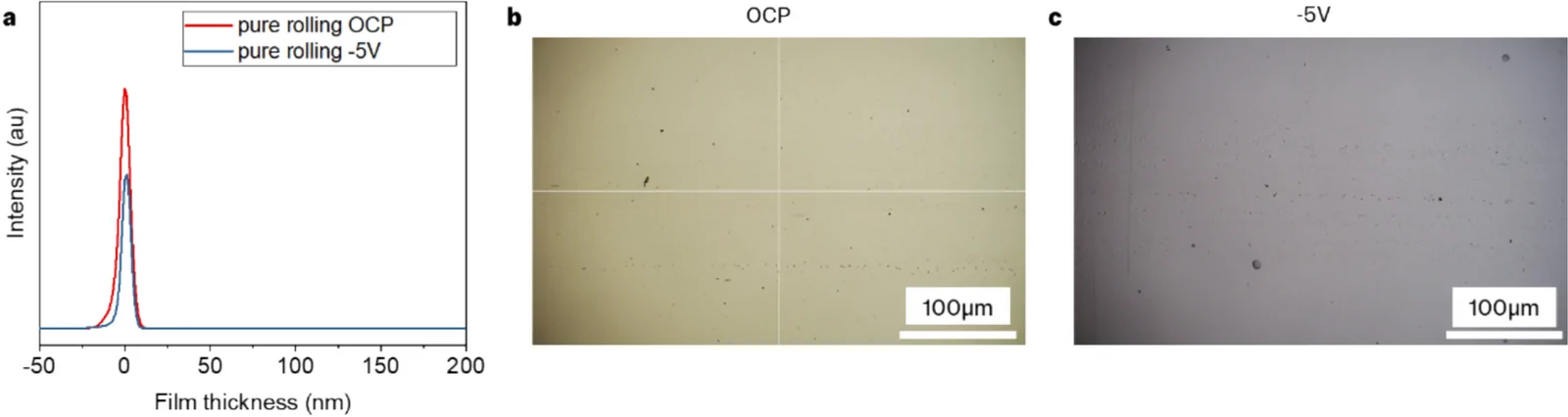
Worn disc surfaces formed under pure rolling condition, i.e. no sliding. **a**. Film thickness distribution at OCP and − 5 V. Optical microscopy images obtained at **b** OCP and **c** − 5 V

The effect of rubbing is further investigated by conducting tests using a MTM-EC tribometer under pure rolling conditions (0% SRR). The load and entrainment speed are as stated in Fig. 1c, and hence, the contact conditions are similar to those above with sliding (SRR = 20%), except now no sliding is involved. Figure 14 shows the worn disc surfaces formed under OCP and − 5 V in pure rolling condition, together with the corresponding surface morphology presented as histograms. The results indicate that the application of a negative voltage, in the absence of sliding, is not sufficient to promote noticeable tribofilm growth under the present test conditions.

These results support the proposition that the substantial BEPite tribofilm growth observed during sliding under applied voltage arises from triboelectrochemical processes that occur when mechanical shearing is present. While electrochemical reactions may promote this process through the generation of Fe species, mechanical force plays a decisive role in activating voltage-driven interfacial reactions, which subsequently contribute to tribofilm formation.

## Conclusion

Ashless P-based antiwear additives are more environmentally acceptable alternatives to conventional antiwear additives such as ZDDP. However, they suffer from relatively low tribofilm growth compared to metal-containing P additives or some phosphorus-sulphur- based ones. In this work, the tribofilms of P-based ashless additives formed under electrified conditions have been examined. Under our experimental conditions, tribofilm formation is primarily governed by applied voltage rather than current, with an anodic surface exhibiting faster tribofilm growth and greater film thickness. Chemical analysis suggests that iron participation plays an important role in tribofilm formation. The tribofilm structure consists of an outer layer of organic species and carbon, and an inner layer rich in phosphorus and iron. Compared with the tribofilm formed under OCP, applying − 5 V produces a denser and thicker film, with improved stability. The discovery that under mechanical shear, anodic oxidation and additive decomposition act cooperatively to build dense and stable tribofilms highlights a new electrochemical-tribochemical coupling mechanism. These insights provide a foundation for designing electro-responsive additives and smart lubricants capable of dynamically adapting to next-generation electrified systems.

## Supplementary Information

Below is the link to the electronic supplementary material.Supplementary file1 (PDF 3092 KB)

## Data Availability

The data that support the findings of this study are available from the corresponding author upon reasonable request.

## References

[1] Kliman, G., Stein, J. (1990). Induction motor fault detection via passive current monitoring—a brief survey. In: Proc. 44th Meeting of the Mechanical Failures Prevention Group, pp. 49–65.

[2] Hadden, T., Jiang, J.W., Bilgin, B., Yang, Y., Sathyan, A., Dadkhah, H., Emadi, A. A review of shaft voltages and bearing currents in EV and HEV motors. In: IECON 2016—42nd Annual Conference of the IEEE Industrial Electronics Society, pp. 1578–1583 (2016). 10.1109/IECON.2016.7793357

[3] Sheng, S. Report on wind turbine subsystem reliability-a survey of various databases (presentation). Technical report, National Renewable Energy Lab. (NREL), Golden, CO (USA) (2013). https://docs.nrel.gov/docs/fy13osti/59111.pdf

[4] Yamamoto, Y., Hirano, F.: Scuffing resistance of phosphate esters II: effect of applied voltage. Wear **66**, 77–86 (1981). 10.1016/0043-1648(81)90034-X

[5] Yousuf, A., Spikes, H., Guo, L., Kadiric, A.: Influence of electric potentials on surface damage in rolling-sliding contacts under mixed lubrication. Tribol. Lett. **73**, 45 (2025). 10.1007/s11249-025-01977-2

[6] Cao-Romero-Gallegos, J.A., Farfan-Cabrera, L.I., Erdemir, A., Pascual-Francisco, J.B.: Lubricated sliding wear of gear material under electrification—a new approach to understanding of the influence of shaft currents in the wear of EV transmissions. Wear **523**, 204782 (2023)

[7] Spikes, H.A.: Beyond ZDDP. Lubr. Sci. **20**, 77–78 (2008). 10.1002/ls.60

[8] Gangopadhyay, A.K., Jensen, R.K., Carter, R.O., III., Uy, D., O’Neill, A.E., Simko, S.J., Gao, H., Stockwell, R.T., Phillips, C.B., Graham, M.E.: Development of zero‐phosphorus engine oils. Lubr. Sci. **20**, 163–180 (2008). 10.1002/ls.54

[9] Devlin, M.T., Guevremont, J., Sheets, R., Loper, J., Guinther, G., Thompson, K., Jao, T.C.: Effect of metal‐free phosphorus anti‐wear compounds on passenger car emissions and fuel economy. Lubr. Sci. **20**, 151–161 (2008). 10.1002/ls.44

[10] Spikes, H.: Low‐and zero‐sulphated ash, phosphorus and sulphur anti‐wear additives for engine oils. Lubr. Sci. **20**, 103–136 (2008)

[11] Kim, B. Tribological performance of ashless antiwear additives under extreme pressure conditions. Thesis, 2010. https://mavmatrix.uta.edu/context/materialscieng_dissertations/article/1041/type/native/viewcontent

[12] Guan, B., Pochopien, B.A., Wright, D.S.: The chemistry, mechanism and function of tricresyl phosphate (TCP) as an anti-wear lubricant additive. Lubr. Sci. **28**, 257–265 (2015)

[13] Restuccia, P., Pedretti, E., Benini, F., Loehlé, S., Righi, M.C.: Phosphorus-based lubricant additives on iron with Machine Learning Interatomic Potentials. Appl. Surf. Sci. (2026). 10.1016/j.apsusc.2026.166599

[14] Wang, J., Zheng, J., Wang, J., Yao, X., Xiong, X., Huang, H.: Synergistic tribological performance of phosphorus-and sulfur-based extreme pressure and anti-wear additives. Lubricants **13**(2), 55 (2025)

[15] Chen, Y.L., Ma, R., Dong, J.Y., Han, Y.M., Ma, W.W., Zhang, E.H., Li, W.M.: New insights into the interactions between phosphorus-and sulfur-containing lubricating additives. Tribol. Int. **211**, 110870 (2025)

[16] Chen, F., Lin, H., Xue, Y., Zhou, F., Dai, B., Wang, C., Han, S.: Phosphorus-containing ionic organomolybdenum lubricant additive for enhancing tribological performance of PAO6 base oil. Langmuir **41**(49), 33409–33421 (2025) 41332329 10.1021/acs.langmuir.5c04756

[17] Gangopadhyay, A., Paputa Peck, M.C., Simko, S.J.: Wear control in a lubricated contact through externally applied electric current. Tribol. Trans. **45**, 302–309 (2002). 10.1080/10402000208982553

[18] Peng, Z., Nassif, A., Georgi, F., Montmitonnet, P., Lahouij, I.: Electric polarity: a key factor in lubricated wear of bearing steel. Tribol. Int. **209**, 110748 (2025). 10.1016/j.triboint.2025.110748

[19] Aguilar-Rosas, O.A., Farfan-Cabrera, L.I., Erdemir, A., CaoRomero-Gallegos, J.A.: Electrified four-ball testing-a potential alternative for assessing lubricants (e-fluids) for electric vehicles. Wear **522**, 204676 (2023). 10.1016/j.wear.2023.204676

[20] Farfan-Cabrera, L.I., Hernández-Peña, A., Resendiz-Calderon, C.D., Lee, P., Sanchez, C., Lee, S., Erdemir, A.: Electrified four-ball testing of ZDDP and MoDTC as additives in low-viscosity synthetic oil. Wear **571**, 205835 (2025). 10.1016/j.wear.2025.205835

[21] Farfan-Cabrera, L.I., Lee, S., Skowron, S., Erdemir, A.: Enhancing lubrication of electrified interfaces by inert gas atmosphere. J. Tribol. **147**, 051104 (2024). 10.1115/1.4066649

[22] Blankespoor, R.L.: Electrochemical oxidation of zinc bis(O,O-dialkyl phosphorodithioates-S, S’). Mediation by 1,1’-Bis(methoxycarbonyl)ferrocene. Inorg. Chem. **24**, 1126–1128 (1985). 10.1021/ic00202a003

[23] Stezeryanskii, E.A., Litovchenko, K.I., Kublanovsky, V.S.: Electro-oxidation of zinc diisooctyldithiophosphate. J. Electroanal. Chem. **390**, 143–145 (1995). 10.1016/0022-0728(95)03893-L

[24] Ozimina, D.: The assessment of ZnDTP tribochemical reactivity by electrochemical simulation. Lubr. Sci. **13**, 45–57 (2000). 10.1016/S0167-8922(02)80019-0

[25] Xu, X., Brandon, N., Spikes, H. Study of zinc dialkyldithiophosphate using electrochemical techniques. In: Proc. Leeds-Lyon Symp. Boundary and Mixed Lubrication, Vienna Sept. 2001, pp. 175–181. 10.1002/ls.3010130105

[26] Ali, M.K.A., Zhang, C., Yu, Q., Sun, Y., Zhou, F., Liu, W.: Do electrification-temperature effects deteriorate ZDDP tribofilms in electric vehicles transmission? Insights into antiwear mechanisms using low-saps oils. Wear **564–565**, 205746 (2025). 10.1016/j.wear.2025.205746

[27] Yamamoto, Y., Yagi, J., Higaki, H.: Effect of externally applied electric field on friction and wear characteristics. JSME Int. J. Ser. 3, Vib., Control Eng., Eng. Ind. **35**, 641–646 (1992). 10.1299/jsmec1988.35.641

[28] He, S., Meng, Y., Tian, Y.: Correlation between adsorption/desorption of surfactant and change in friction of stainless steel in aqueous solutions under different electrode potentials. Tribol. Lett. **41**, 485–494 (2011). 10.1007/s11249-010-9604-6

[29] Liu, C., Li, W., Ouyang, C., Tian, Y., Meng, Y.: Voltage-assisted tribofilm formation of sulfur-and phosphorus-free organic molybdenum additive on bearing steel surfaces in industrial base oils. Tribol. Lett. **70**, 19 (2022). 10.1007/s11249-022-01562-x

[30] Ali, M.K.A., Sun, Y., Zhang, C., Yu, Q., Zhao, C., Zhou, F., Liu, W.: Improving tribological performance of electrified steel interfaces in e-mobility systems using ash-sulfur-less oil additives based on amine salts-phosphoric esters. Tribol. Int. **205**, 110561 (2025)

[31] Endo, K., Fukuda, Y., Takamiya, O.: Wear behaviours of metals under lubricated conditions and the effects of small electric potential. Trans. Jpn. Soc. Mech. Eng. **14**, 1281–1288 (1971)

[32] He, Y., Luo, J., Xie, G.: Characteristics of thin liquid film under an external electric field. Tribol. Int. **40**(10–12), 1718–1723 (2007)

[33] Głogowski, M., Smykowski, D., Pietrowicz, S.: The effect of an electric field on the sliding friction of the silicone rubber against selected metals in motor base oils. Energies **16**(9), 3954 (2023)

[34] Shor, G.I., Blagovidov, I., Eustigneev, E., Lapin, V.P.: Antiwear and antiscuff properties of lubricating oils in relation to electric potential at metal-oil phase boundary. Chem. Tech. Fuels Oil **8**, 770–772 (1972)

[35] Lu, R., Kawada, S., Tani, H., Koganezawa, S.: The influence of electric current on the friction behavior of lubricant molecules. Tribology Online **18**(3), 83–90 (2023)

[36] Xie, G., Luo, J., Guo, D., Liu, S., Li, G.: Damages on the lubricated surfaces in bearings under the influence of weak electrical currents. Sci. China Technol. Sci. **56**(12), 2979–2987 (2013)

[37] Yang, Q., Duan, F.: Tribological properties of phosphate ester confined between iron-based surfaces. Langmuir **40**(7), 3738–3747 (2024) 10.1021/acs.langmuir.3c03464 38332575

[38] Sharma, V., Gabler, C., Doerr, N., Aswath, P.B.: Mechanism of tribofilm formation with P and S containing ionic liquids. Tribol. Int. **92**, 353–364 (2015)

[39] Luiz, J.F., Spikes, H.A.: Tribofilm formation, friction and wear-reducing properties of some phosphorus-containing antiwear additives. Tribol. Lett. **68**(3), 75 (2020)

[40] Hamrock, B.J. and Dowson, D., 1981. Ball bearing lubrication: the elastohydrodynamics of elliptical contacts. Ball bearing lubrication: The elastohydrodynamics of elliptical contacts by Hamrock, pp.41687.

[41] American Petroleum Institute: Engine Oil Licensing and Certification System Seventeenth Edition. API 1509 Appendix E (2021)

[42] Ali, M.K.A., Xianjun, H.: Exploring the lubrication mechanism of CeO2 nanoparticles dispersed in engine oil by bis (2-ethylhexyl) phosphate as a novel antiwear additive. Tribol. Int. **165**, 107321 (2022)

[43] Zhao, B., Zhang, M., Zhang, B., Mou, Z., Tang, W., Wang, Z., Wang, B.: Synthesis of bis (2-ethylhexyl) phosphate modified carbon quantum dots for enhanced oil-based lubrication. N. J. Chem. **50**(3), 1452–1459 (2026)

[44] Benedet, J., Green, J.H., Lamb, G.D., Spikes, H.A.: Spurious mild wear measurement using white light interference microscopy in the presence of antiwear films. Tribol. Trans. **52**, 841–846 (2009)

[45] Benedet, J. F. L. (2012). Low and Zero SAPS Antiwear Additives for Engine Oils. PhD Thesis, Imperial College London

[46] Lorenz, M., Pawlicki, A.A., Hysmith, H.E., Cogen, K., Thaker, H., Ovchinnikova, O.S.: Direct multimodal nanoscale visualization of early phosphorus-based antiwear tribofilm formation. ACS Appl. Mater. Interfaces **14**(30), 35157–35166 (2022) 35862906 10.1021/acsami.1c16761

[47] Xia, Y., Zhang, S., Wang, Z., Zhang, C.: New insight into tribofilm composition and structure of iron polyphosphate-rich tribofilm from formulated engine oil by ToF-SIMS. J. Mater. Sci. **59**(21), 9533–9546 (2024)

[48] Yang, C., Guo, Y., Zhang, H., Guo, X.: Utilization of electric fields to modulate molecular activities on the nanoscale: from physical properties to chemical reactions. Chem. Rev. **125**(1), 223–293 (2024) 39621876 10.1021/acs.chemrev.4c00327

[49] Zhu, Z., Ewen, J.P., Kritikos, E.M., Giusti, A., Dini, D.: Effect of electric fields on the decomposition of phosphate esters. J. Phys. Chem. C **128**(38), 15959–15973 (2024) 10.1021/acs.jpcc.4c04412 PMC11440609 39355011

[50] Spikes, H.: Mechanisms of ZDDP—an update. Tribol. Lett. **73**(1), 38 (2025)

[51] Saba, C.S., Forster, N.H.: Reactions of aromatic phosphate esters with metals and their oxides. Tribol. Lett. **12**(2), 135–146 (2002)

