# Supplementary material for: Voltage-Driven Growth of Phosphorus Tribofilms
Source: Tribol Lett. 2026 Jul 8;74(3):79. doi: 10.1007/s11249-026-02173-6 (PMC13341739; doi:10.1007/s11249-026-02173-6)
Supplement: Supplementary file 1 — Supplementary file1 (PDF 3092 KB) [file 11249_2026_2173_MOESM1_ESM.pdf]

Voltage-Driven Growth of Phosphorus Tribofilms

Yun Zhao[a], Jie Zhang[a], Hugh A. Spikes[a], & Janet S.S. Wong[a,*]

[a] Department of Mechanical Engineering, Imperial College London, London SW7 2AZ, UK

*Email: j.wong@imperial.ac.uk (Janet S.S. Wong)

S1: Estimation of lubrication film thickness and Lambda ratio

The ball roughness R_{q1} is 8.2nm and disc roughness R_{q2} is 6nm, so the composite surface roughness is 10.17 nm. EHD central film thickness h_0 is 3.35nm calculated based on Hamrock & Dowson equation.

$$\frac{h_0}{R_x} = 2.69\bar{U}^{0.67}\bar{G}^{0.53}\bar{W}^{-0.067}\left(1 - 0.61e^{-0.75(R_y/R_x)^{0.64}}\right)$$

$$\bar{U} = \frac{U\eta_0}{E'R_x}$$

$$\bar{G} = \alpha E'$$

$$\bar{W} = \frac{W}{E'R_x^2}$$

$$\frac{2}{E'} = \frac{(1-v_1^2)}{E_1} + \frac{(1-v_2^2)}{E_2}$$

$$\lambda = \frac{h_0}{R_{qc}}$$

$$R_{qc} = \sqrt{R_{q1}^2 + R_{q2}^2}$$

$$\frac{2}{E'} = \frac{(1-v_1^2)}{E_1} + \frac{(1-v_2^2)}{E_2}$$

h_0 is estimated based on an average speed of U=0.05m/s, viscosity η_0 at 80°C is 1.7cP, $E_1 = E_2$ = 207GPa, R_x = 0.009525 (3/4 ball), $v_1 = v_2 = 0.293$, pressure viscosity coefficient α = 14 GPa^{-1}, normal load W=31 N.

S2: Estimation of heat generation

Joule heating:

Current I = 0.5 mA, resistance R = average voltage/5V x 1MΩ = 2.32 x 10^5 Ω

Power $Q_{sliding}$ = I^2R = 58 mW

Frictional heating:

Friction coefficient μ = 0.11, normal load W = 31N, SRR = 20%, entrainment speed = 50mm/s, sliding speed U = 55–45mm/s = 10mm/s = 0.01m/s

Power $P_{current}$ = μWU = 0.11 × 31 × 0.01 = 34mW

Table S1. Parameters and values used for thermal generation calculations

Sliding/rolling ratio	SRR	0.2	
Entrainment speed	U	0.05	m/s

Sliding speed	u_s	0.01	m/s
Load	W	31	N
Semi contact width	a	124.7620594	um
Maximum pressure	p_{max}	9.51E+08	Gpa
Film thickness	h	3	nm
Friction coefficient	m_u	0.11	
Shear stress	τ_{mean}	69.7331745	Mpa
	q-dot	697331.745	W/m^2
Thermal conductivity	K	28	J/msK
Density	r	7810	kg/m^3
Specific heat	c	485	J/kgK
	K_{rc}	106059800	
Oil thermal conductivity	K_{oil}	0.125	W/mK
	J	168778.5596	ub/x
	x	7.39206E-06	K/rc

Flash temperature calculation between two rubbing surfaces:

$\Delta\theta = 9/32*1/2*Q/aK$

Flash temperature by sliding $\Delta_{\theta surface,sliding}$ = 2.7K

Flash temperature by current $\Delta_{\theta surface,current}$ = 4.7K

Flash temperature calculation in the lubricant film:

$\Delta\theta=1/(8K_{oil})*\tau*us*h = 1/(8K_{oil})*Q*h/\pi a^2$

Flash temperature by sliding $\Delta_{\theta oil,sliding}$ = 2.1K

Flash temperature by current $\Delta_{\theta oil,current}$ = 3.6K

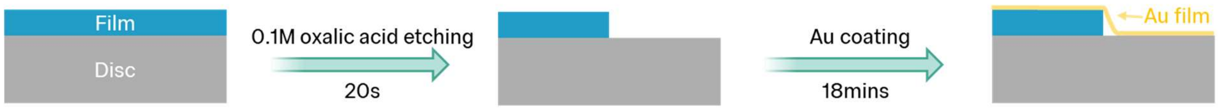

Figure S1. Tribofilm etching process for film thickness measurement.

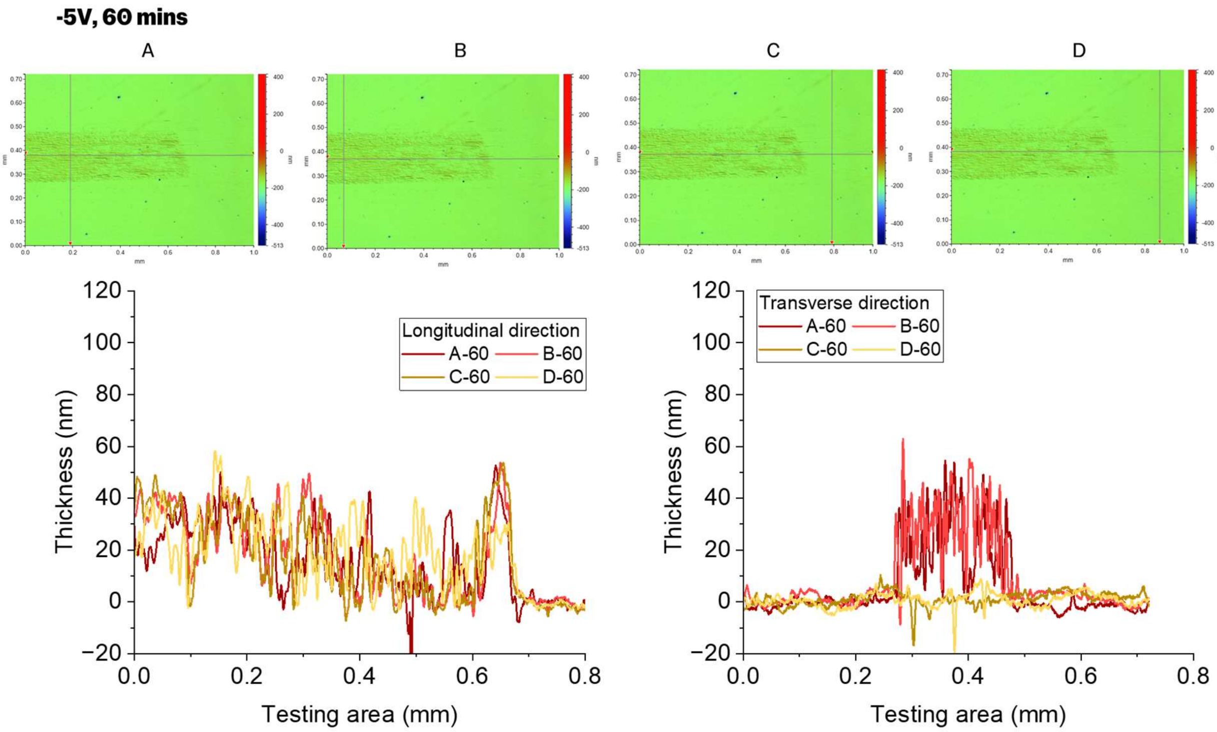

Figure S2. Tribofilm results of BEPite in PAO2 rubbing for 60 mins under -5V.

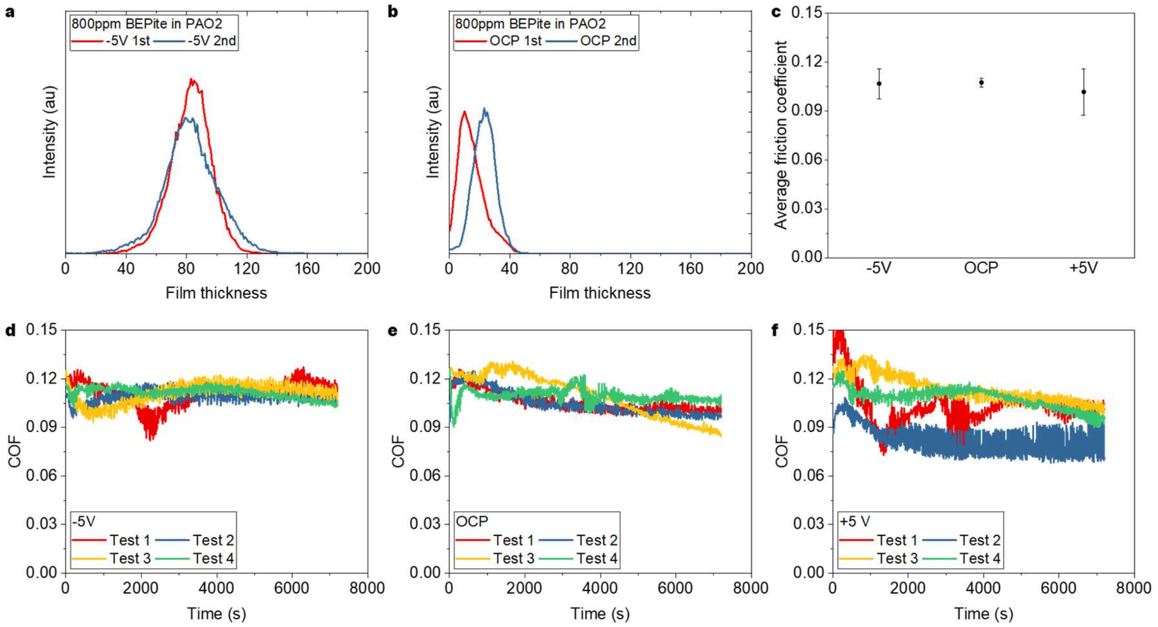

Figure S3. Tribological performance of 800 ppm BEPite in PAO2 under different applied voltages. **a.** film thickness distribution at -5V. The testing region is 50 μm x 100 μm. **b.** film thickness distribution at OCP. **c.** Average friction coefficient from curves in **d**, **e**, and **f**. They are average values by summing the corresponding data points and dividing by the number of data points. The final mean value was then obtained by averaging the four group means, and the error bar represents the standard deviation among these four means. The average COF

values of the four curves in **d** are 0.112, 0.109, 0.111, and 0.111, respectively. The corresponding values in **e** are 0.106, 0.105, 0.110, and 0.110, respectively, while those in **f** are 0.103, 0.0819, 0.114, and 0.108, respectively.

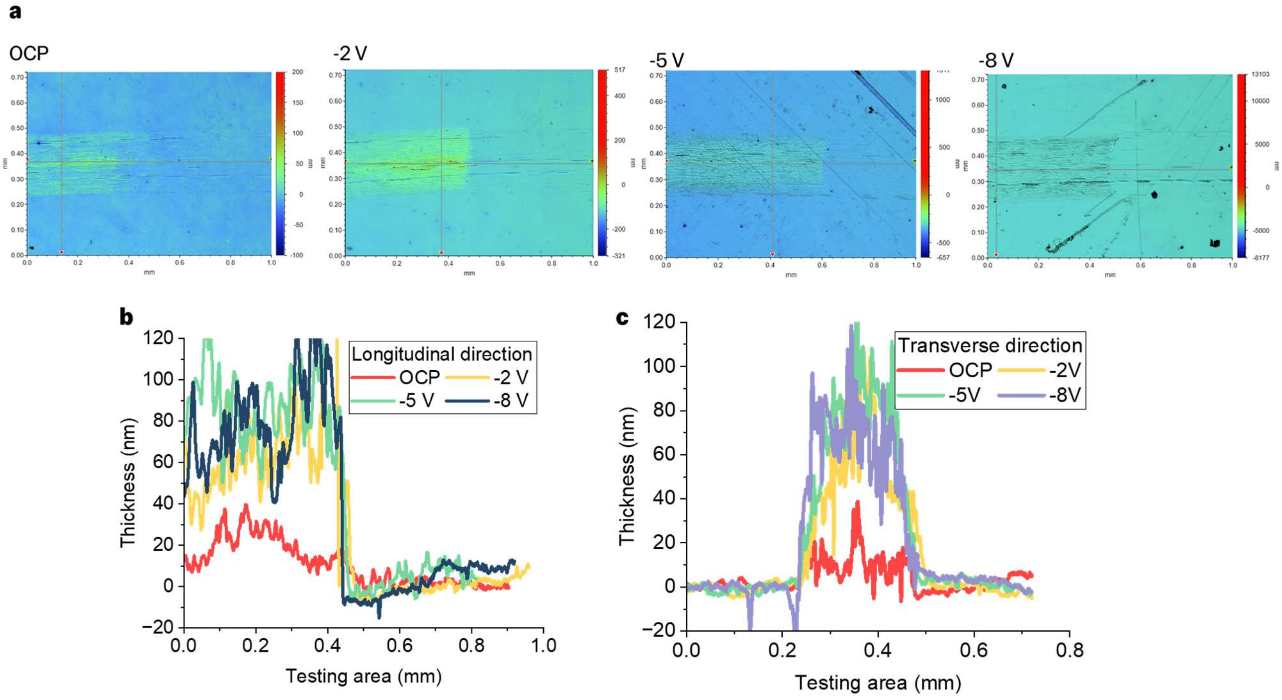

Figure S4. Tribofilm thickness profiles across the wear track formed under different applied voltages.

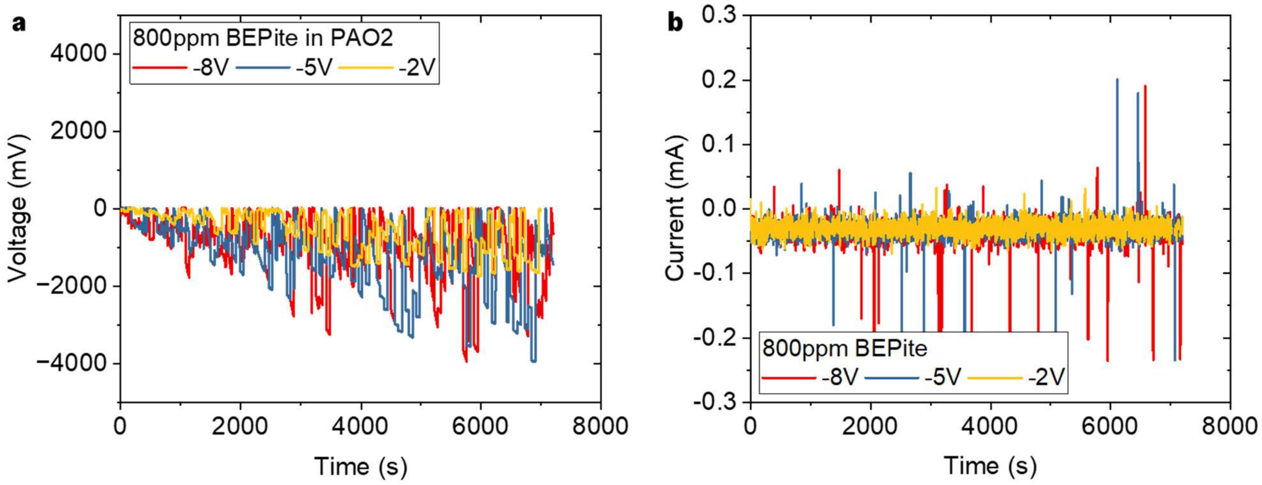

Figure S5. Real-time (**a**) voltage and (**b**) current recorded under various applied voltage conditions.

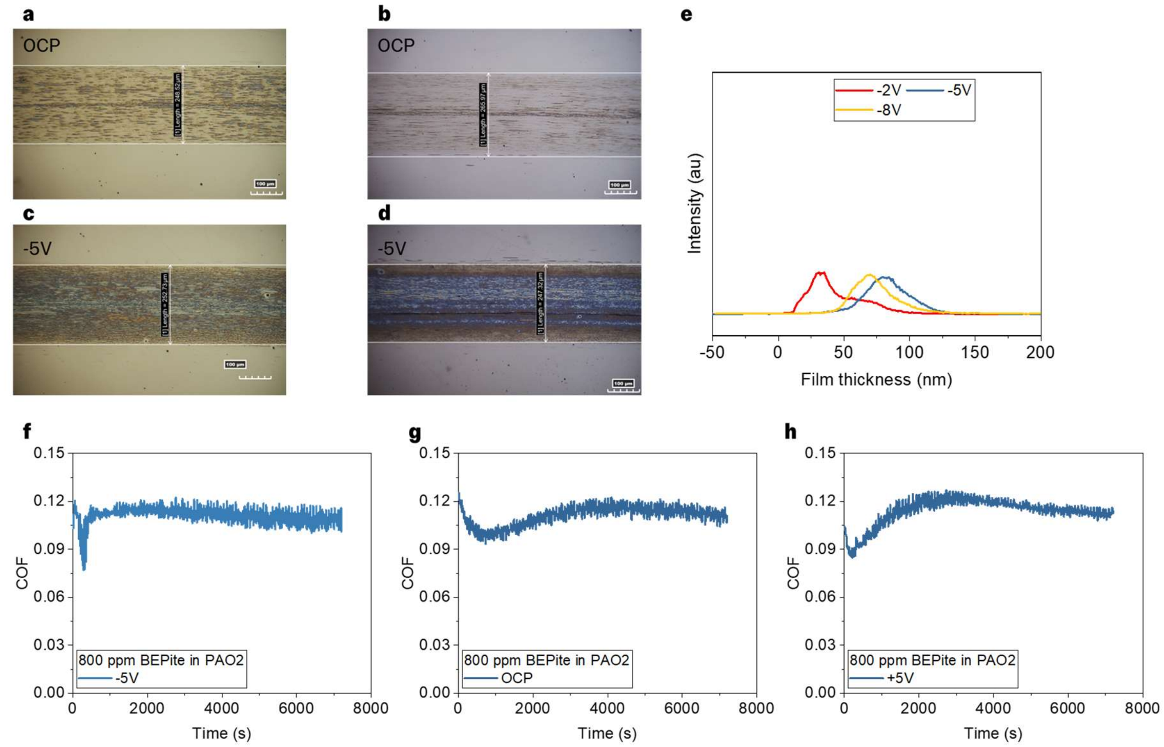

Figure S6. Two sets of repeat tests: (**a**, **b**) optical microscope images of wear tracks generated under OCP conditions, and (**c**, **d**) wear tracks generated under -5 V. **e.** Film thickness distribution at -2V, -5V, and -8V. **f-h**. COF curves under different applied voltage conditions.

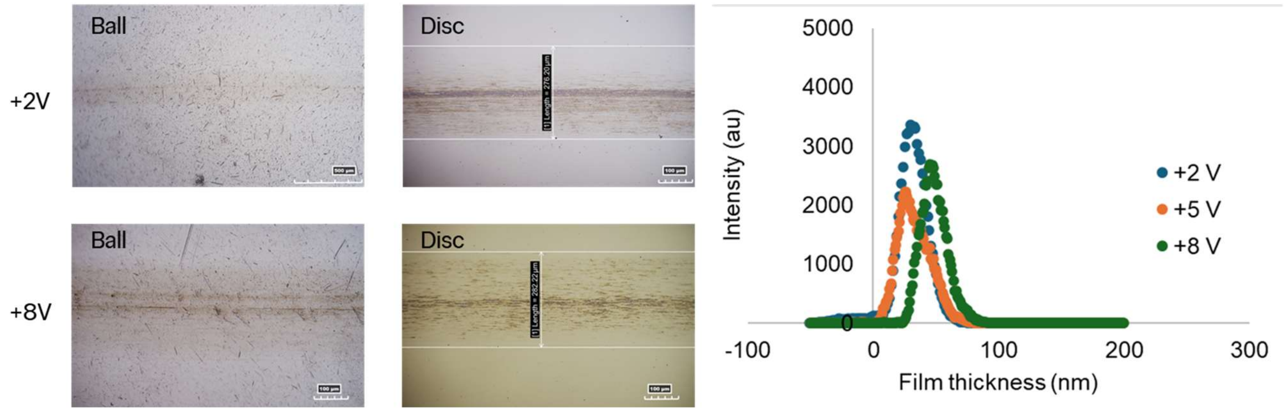

Figure S7. Optical microscope images of the ball and disc surfaces under positive voltage conditions and the corresponding film thickness distribution.

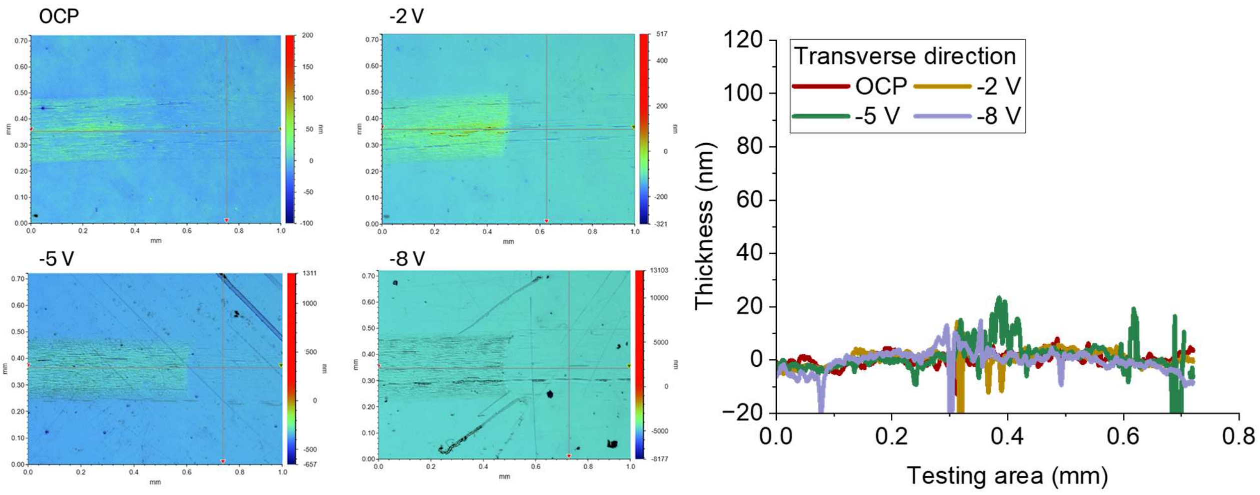

Figure S8. Steel surfaces underneath BEPite tribofilms formed in PAO2 after rubbing for 120 mins under applied voltage conditions.

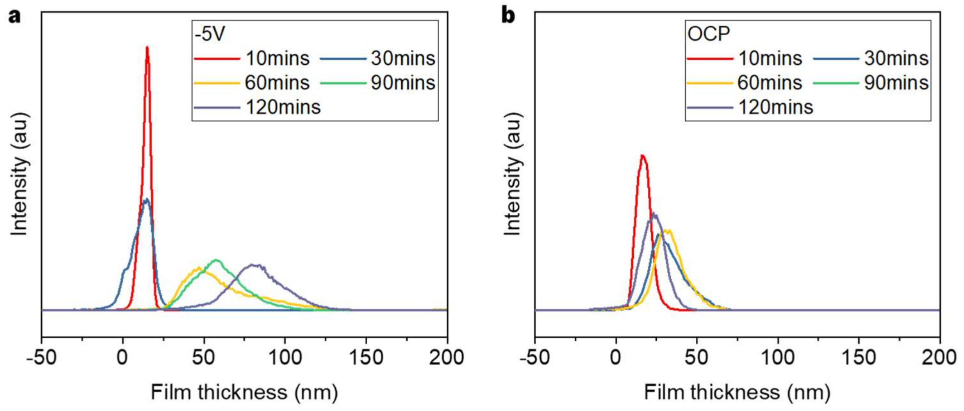

Figure S9. BEPite tribofilm thickness distribution on anodic discs over different rubbing durations under (a) -5V and (b) OCP.

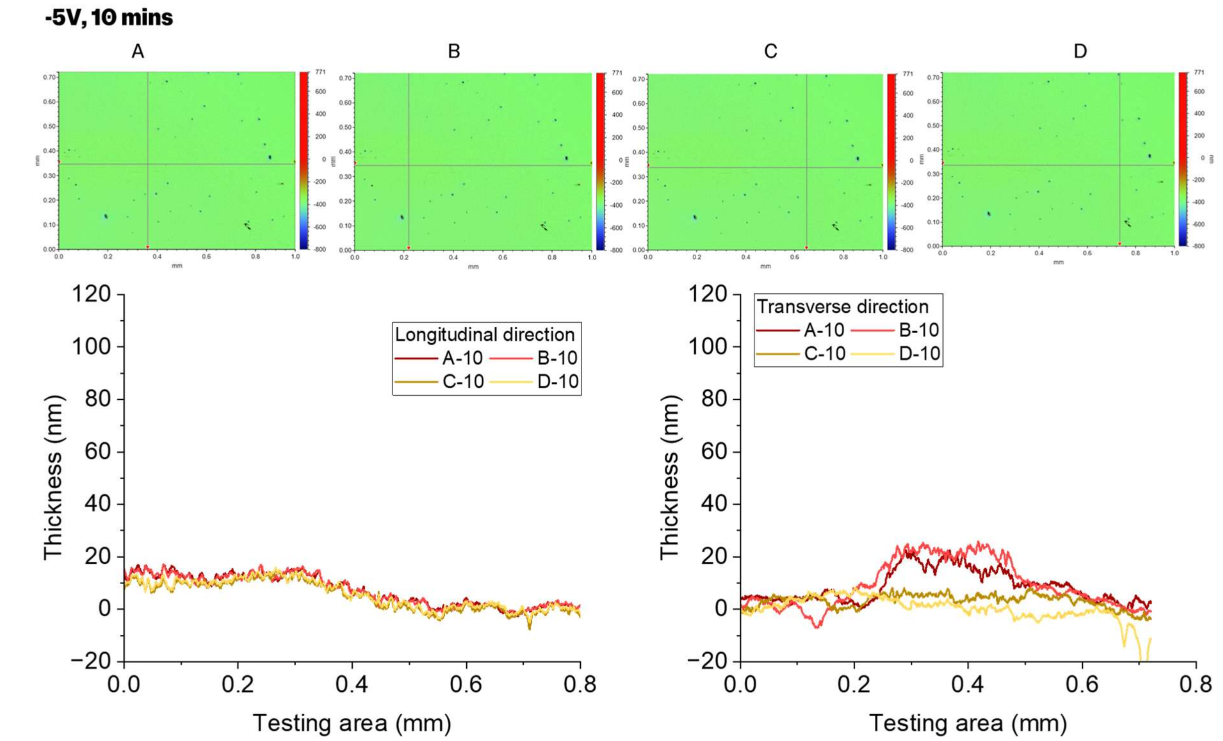

Figure S10. Tribofilm results of BEPite in PAO2 rubbing for 10 mins under -5V.

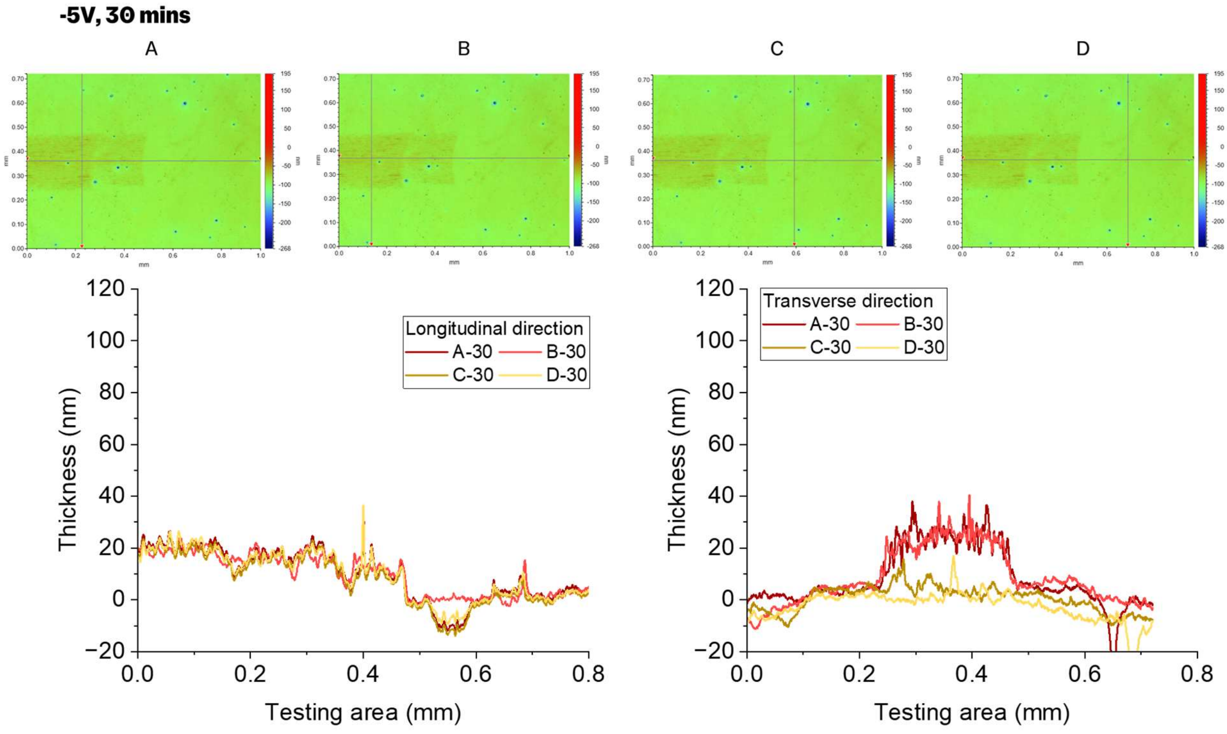

Figure S11. Tribofilm results of BEPite in PAO2 rubbing for 30 mins under -5V.

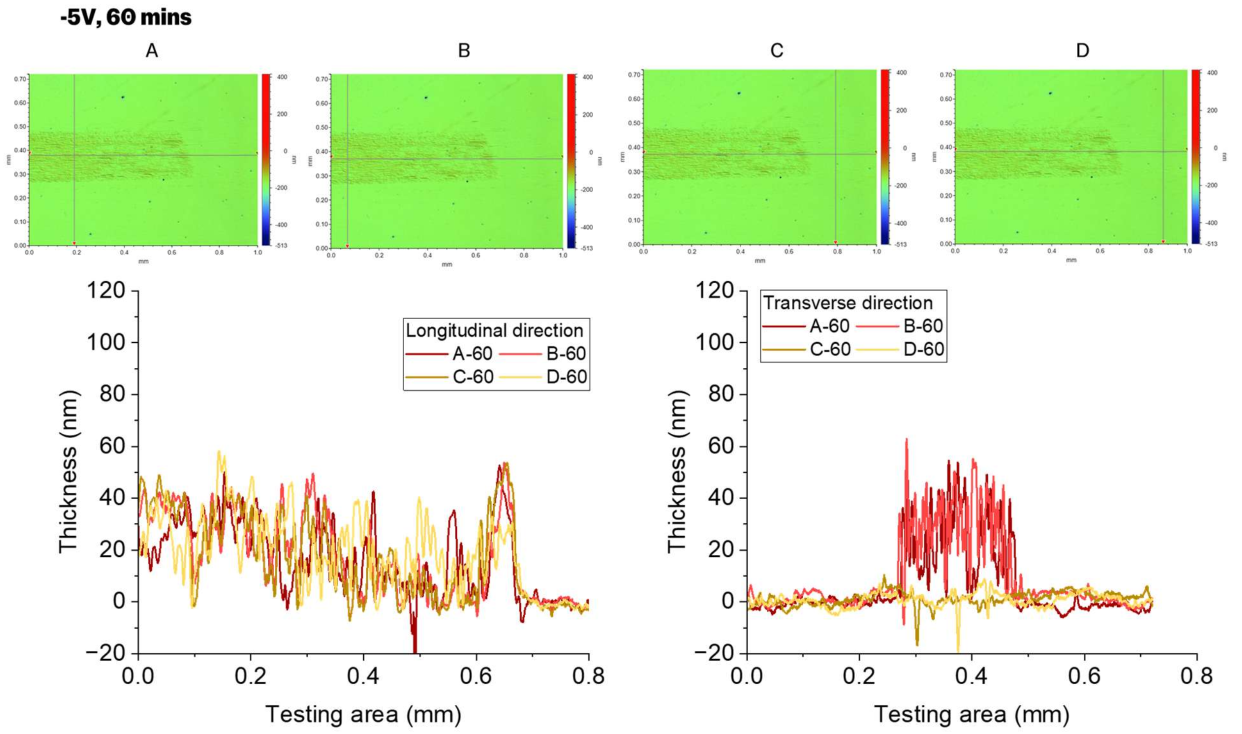

Figure S12. Tribofilm results of BEPite in PAO2 rubbing for 60 mins under -5V.

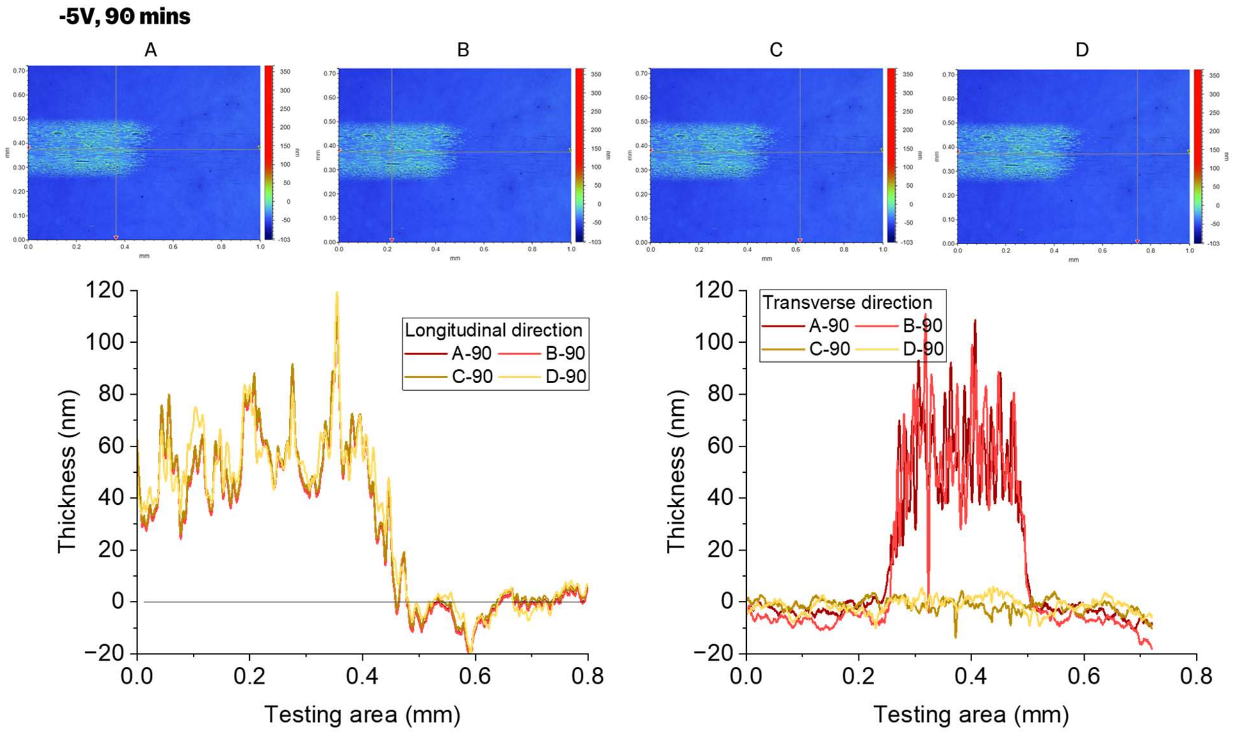

Figure S13. Tribofilm results of BEPite in PAO2 rubbing for 90 mins under -5V.

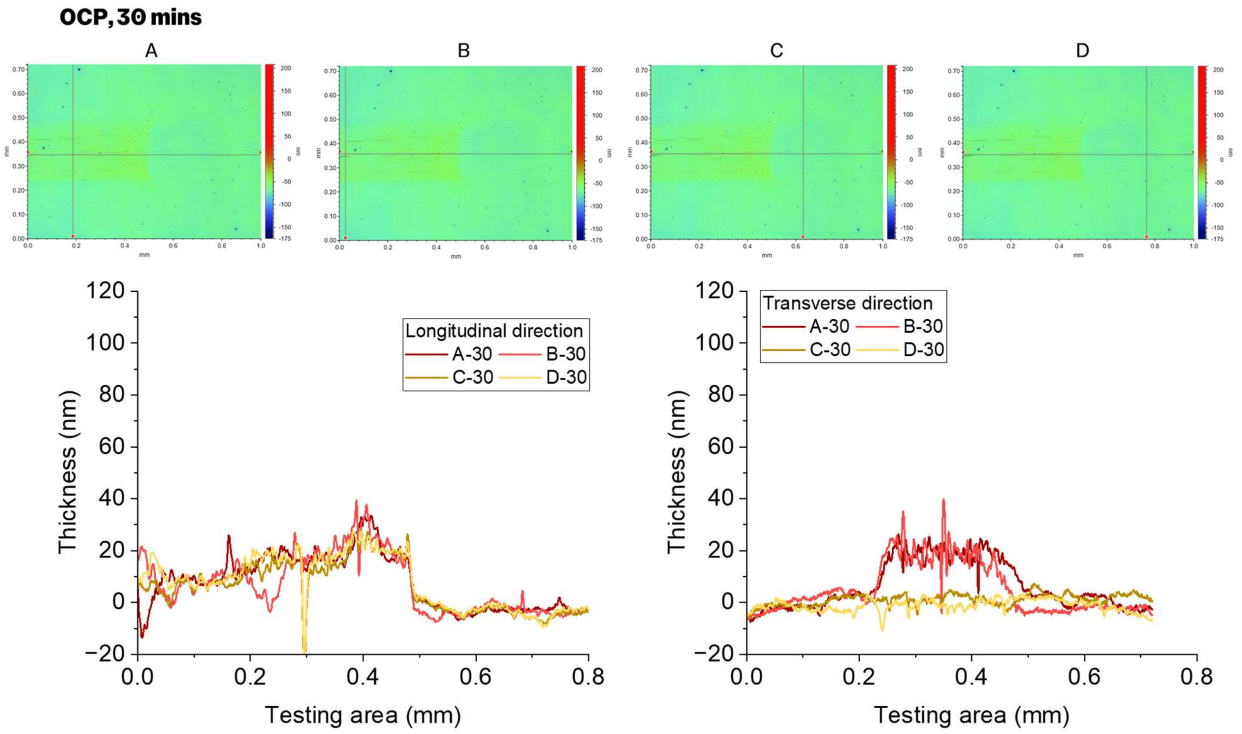

Figure S14. Tribofilm results of BEPite in PAO2 rubbing for 30 mins under OCP.

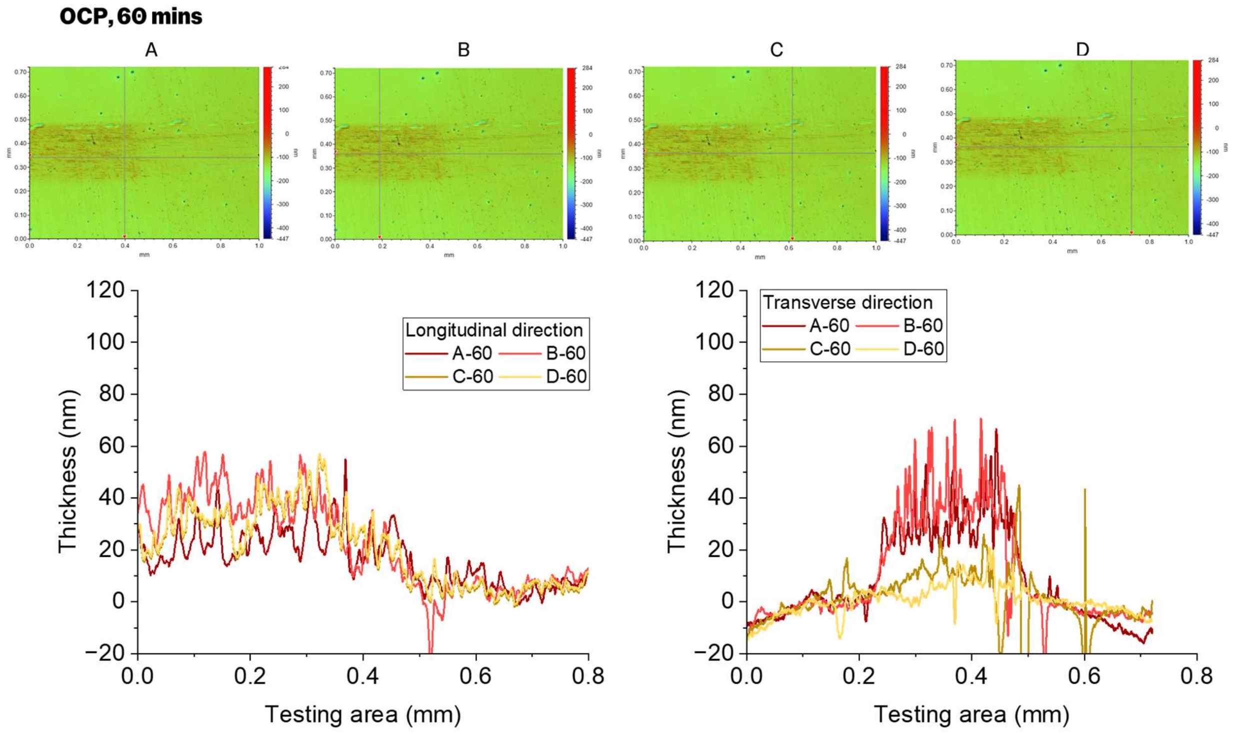

Figure S15. Tribofilm results of BEPite in PAO2 rubbing for 60 mins under OCP.

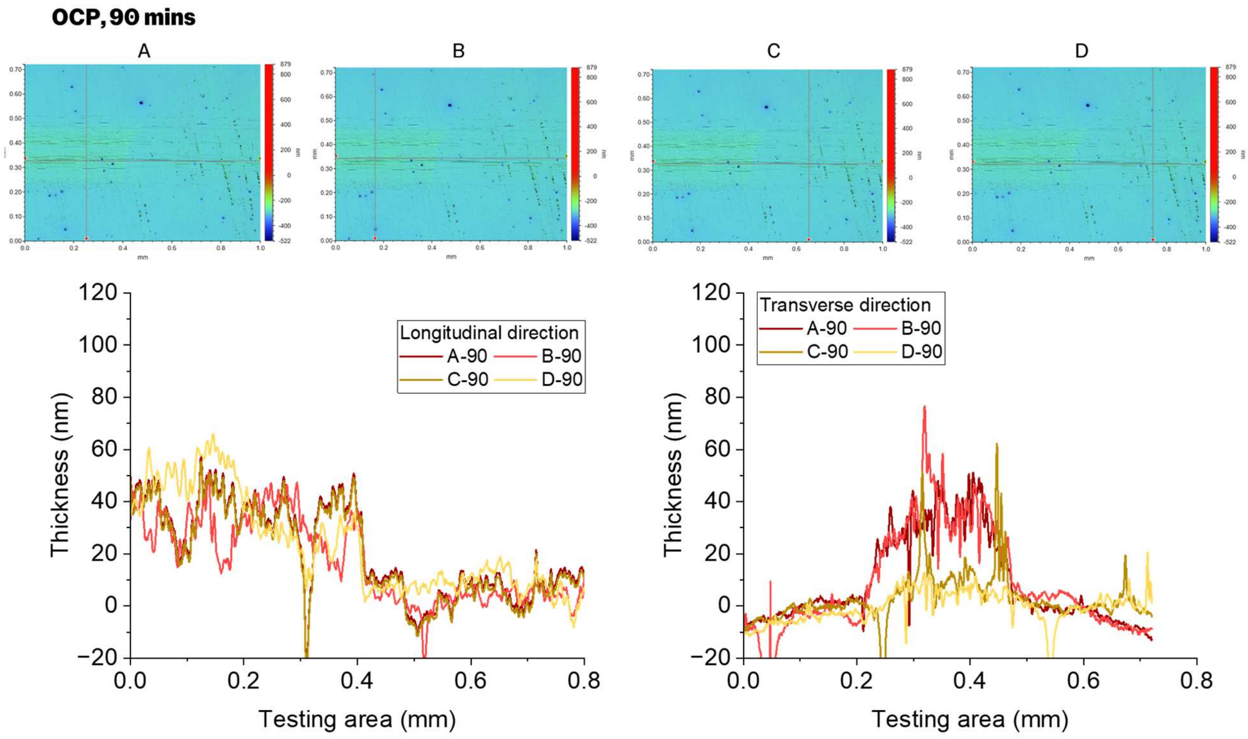

Figure S16. Tribofilm results of BEPite in PAO2 rubbing for 90 mins under OCP.

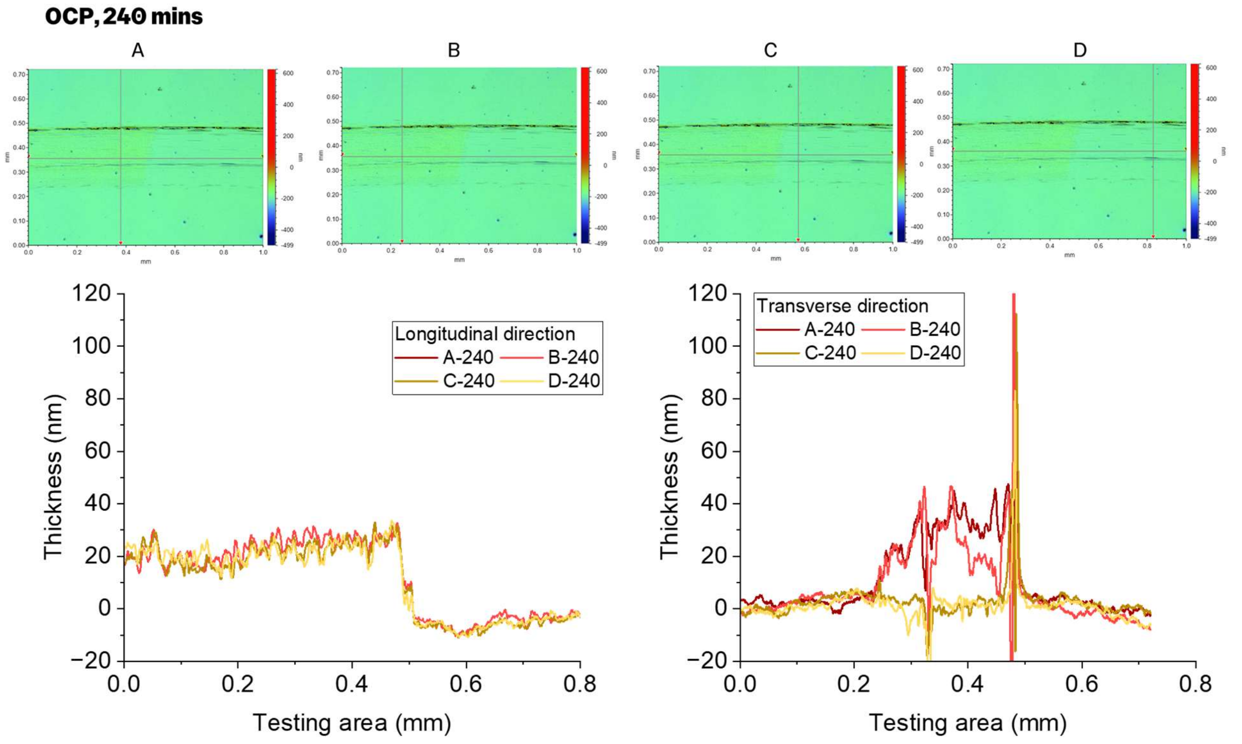

Figure S17. Tribofilm results of BEPite in PAO2 rubbing for 240 mins under OCP.

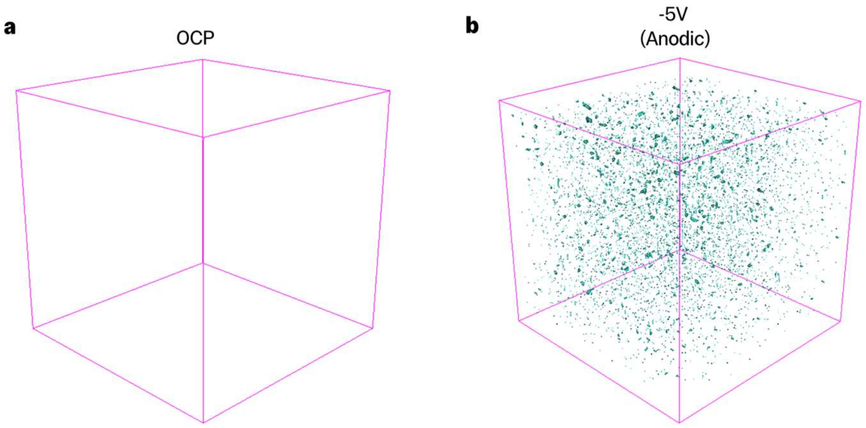

Figure S18. 3D TOF-SIMS tomographic reconstruction of PO_4^- distribution of discs treated at OCP (**a**) and -5V (**b**).

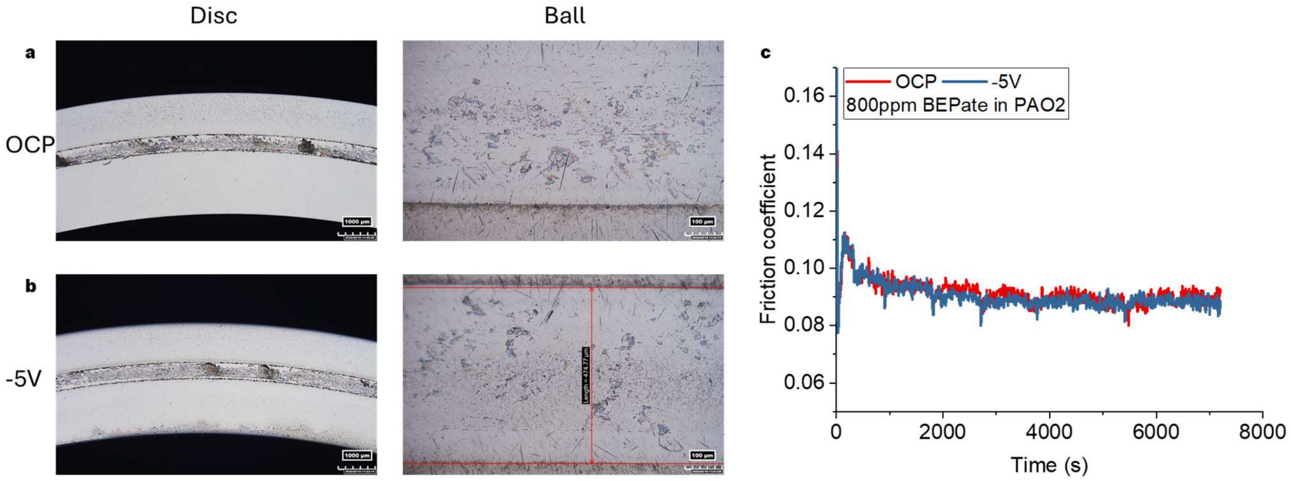

Figure S19. a. Wear track of diisopropyl phosphite in PAO2 at OCP. **b.** Wear track of diisopropyl phosphite in PAO2 at -5V. **c.** COF results of diisopropyl phosphite in PAO2.

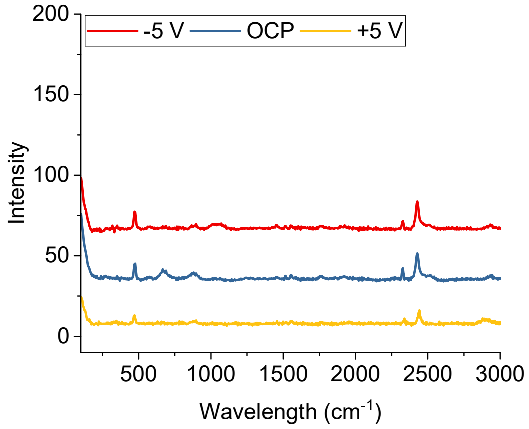

Figure S20. Raman spectroscopy of discs treated at applied voltage conditions.

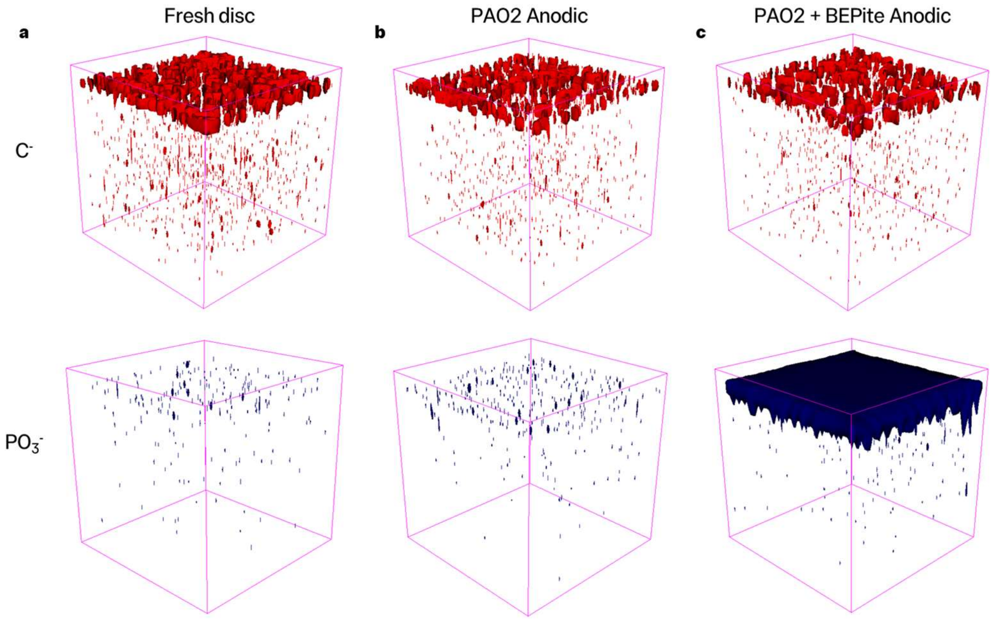

Figure S21. 3D TOF-SIMS tomographic reconstruction of C^- and PO_3^- distribution of discs. **a.** Fresh disc. **b.** Disc treated in PAO2 at -5 V. **c.** Disc treated in BEPite in PAO2 at -5 V.

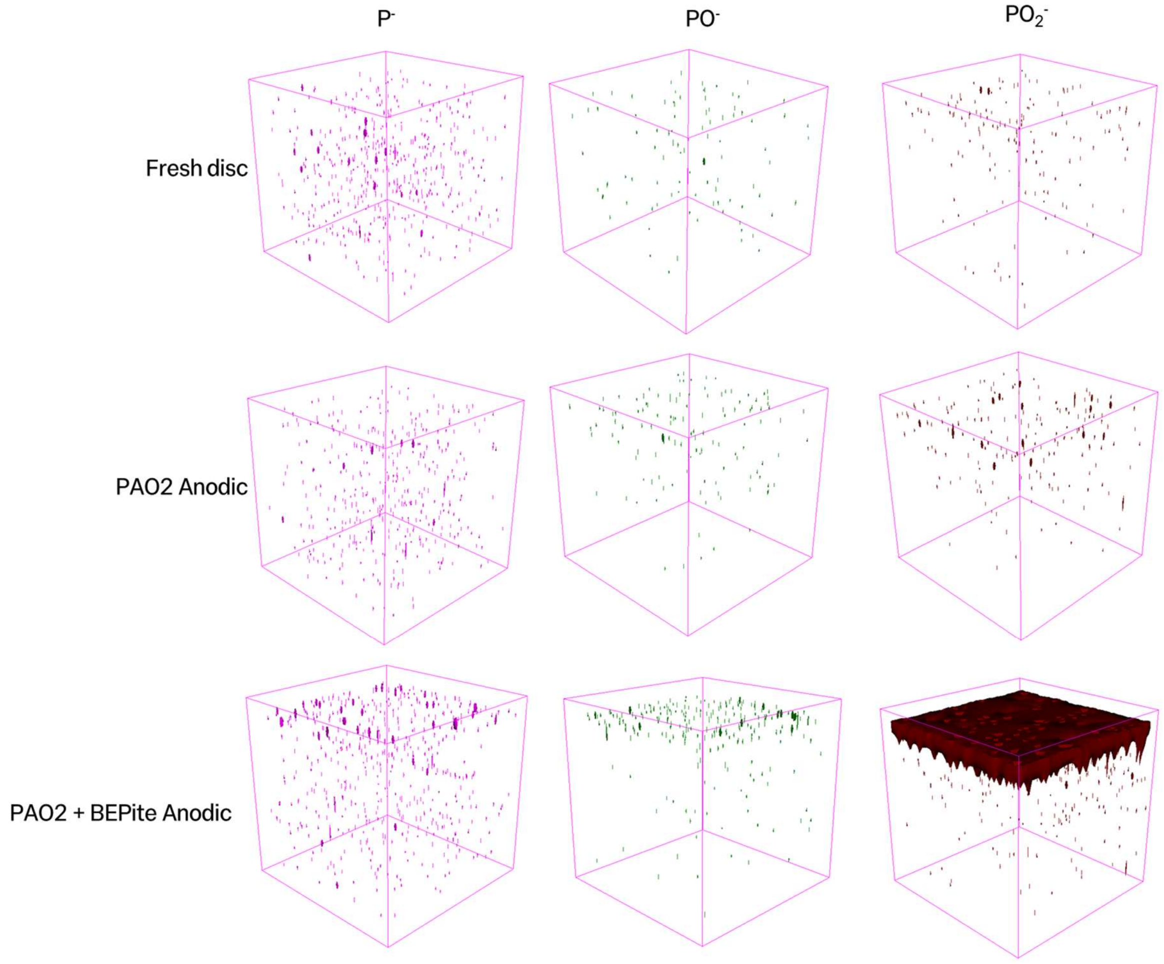

Figure S22. 3D TOF-SIMS tomographic reconstruction of P^-, PO^- and PO_2^- distribution of discs.
